# Selection on the promoter regions plays an important role in complex traits during duck domestication

**DOI:** 10.1186/s12915-023-01801-0

**Published:** 2023-12-21

**Authors:** Zhong-Tao Yin, Xiao-Qin Li, Yun-Xiao Sun, Jacqueline Smith, Maxwell Hincke, Ning Yang, Zhuo-Cheng Hou

**Affiliations:** 1 National Engineering Laboratory for Animal Breeding and Key Laboratory of Animal Genetics, Breeding and Reproduction, College of Animal Science and Technology, MARA, China Agricultural University, No. 2 Yuanmingyuan West Rd, Beijing, 100193 China; 2 The Roslin Institute & R(D)SVS, University of Edinburgh, Easter Bush, Midlothian, EH25 9RG UK; 3 Faculty of Medicine, University of Ottawa, 451 Smyth Road, Ottawa, ON K1H 8M5 Canada

**Keywords:** Promoter, Accumulated variants, Gene expression, Domestication, Duck

## Abstract

**Background:**

Identifying the key factors that underlie complex traits during domestication is a great challenge for evolutionary and biological studies. In addition to the protein-coding region differences caused by variants, a large number of variants are located in the noncoding regions containing multiple types of regulatory elements. However, the roles of accumulated variants in gene regulatory elements during duck domestication and economic trait improvement are poorly understood.

**Results:**

We constructed a genomics, transcriptomics, and epigenomics map of the duck genome and assessed the evolutionary forces that have been in play across the whole genome during domestication. In total, 304 (42.94%) gene promoters have been specifically selected in Pekin duck among all selected genes. Joint multi-omics analysis reveals that 218 genes (72.01%) with selected promoters are located in open and active chromatin, and 267 genes (87.83%) with selected promoters were highly and differentially expressed in domestic trait-related tissues. One important candidate gene *ELOVL3*, with a strong signature of differentiation on the core promoter region, is known to regulate fatty acid elongation. Functional experiments showed that the nearly fixed variants in the top selected *ELOVL3* promoter in Pekin duck decreased binding ability with *HLF* and increased gene expression, with the overexpression of *ELOVL3* able to increase lipid deposition and unsaturated fatty acid enrichment.

**Conclusions:**

This study presents genome resequencing, RNA-Seq, Hi-C, and ATAC-Seq data of mallard and Pekin duck, showing that selection of the gene promoter region plays an important role in gene expression and phenotypic changes during domestication and highlights that the variants of the *ELOVL3* promoter may have multiple effects on fat and long-chain fatty acid content in ducks.

**Supplementary Information:**

The online version contains supplementary material available at 10.1186/s12915-023-01801-0.

## Background

Domestic animals have experienced notable phenotypic changes under strong artificial selection over evolutionarily short periods of domestication and are thus important models for studying positive selection for complex traits [1–4]. Studies show that the improvement of quantitative traits such as body weight, muscle weight, and fat content of domestic animals is accompanied by the continuous selection altering the allele frequency of variants [5–8]. At present, identifying the genes and variants that underlie complex developmental and agricultural traits is a great challenge for quantitative genetics and modern breeding [6, 9], and the genetic mechanisms that regulate complex quantitative traits during domestication are not clear.

To date, extensive studies have been conducted on the genetic basis of phenotypic changes in domesticated species [10–12]. Researchers have found that some missense mutations in the coding regions of genes can cause the observed phenotypic differences; however, most traits of economic importance in domesticated animals and of medical importance in humans are quantitative in nature, and a proportion of quantitative variants are still unexplained [13–17]. Recently, researchers have explored the effect of artificial selection for domestication traits in genome regulatory regions [18–20]. The promoter is an important regulatory region in the genome that affects the expression of protein-coding genes. Some studies have shown that a large number of variants are located in gene promoters and demonstrated the regulatory effect of variation on gene expression and traits [18, 21–23]. Although variants in gene promoter regions have been shown to have important effects on quantitative traits, few studies have been conducted to explore the genome-wide patterns and roles of promoters in duck domestication and economic trait improvement.

Duck (*Anas platyrhynchos*) is one of the most successfully domesticated birds and provides a major source of quality meat, eggs, and down feathers for humans. Studies have shown that most domestic duck breeds originated from the mallard about 2200–2500 years ago [24, 25]. As a world-standard domesticated breed, the Pekin duck is famous for its white feathers, large body size, plump breast muscles, superior adipose deposition, and high yield of eggs. Comparison of Pekin duck and its wild ancestor will provide an excellent model for the study of quantitative trait improvement under artificial selection [25]. In previous work, we have assembled high-quality reference genomes of mallard and Pekin duck, providing chromosome-level genome sequence and gene annotation [26, 27], which enables us to comprehensively use multi-omics data to study the molecular mechanism of complex trait changes during duck domestication.

In this study, we combined large-scale genome resequencing, RNA-Seq, Hi-C and ATAC-Seq analysis, and functional experiments to assess the role of selection on gene promoters during duck domestication. A multi-omics map was constructed, including 12.6 million single-nucleotide polymorphisms (SNPs), 3 million insertions/deletions (InDels), 74,490 structural variants (SVs), 249,326 potential regulatory elements, over 1000 topologically associating domains (TAD), and gene expression levels of 16 tissues, from which we demonstrate the important role of gene promoter selection for complex traits during domestication. Finally, we demonstrate that the strong differentiation within the *ELOVL3* promoter, which is the key gene regulating high-fat content and unsaturated fatty acid in birds, has resulted in several variants that are nearly fixed in Pekin ducks and show increased expression in Pekin duck liver, as confirmed with in vitro mutation experiments. We detected around a 50% increase in fat deposition and a 39% increase in unsaturated fatty acid content in cells that overexpress *ELOVL3*. Our results shed new light on the genetic mechanisms that underlie domestication and modern breeding in Pekin ducks, with important implications for the future improvement of this important species.

## Results

### Identification and characterization of genetic variants during duck domestication

We sequenced 45 Pekin ducks (coverage, 12–21X; average, 16X), combined with the genome resequencing data of 40 mallards from public databases [24] to detect variants in duck populations (Table S1). The sequencing reads were aligned to the mallard reference genome [26] (GCA_008746955.1) using BWA, with the mapped rate ranging from 98.33 to 99.47%.

**Fig. 1:**
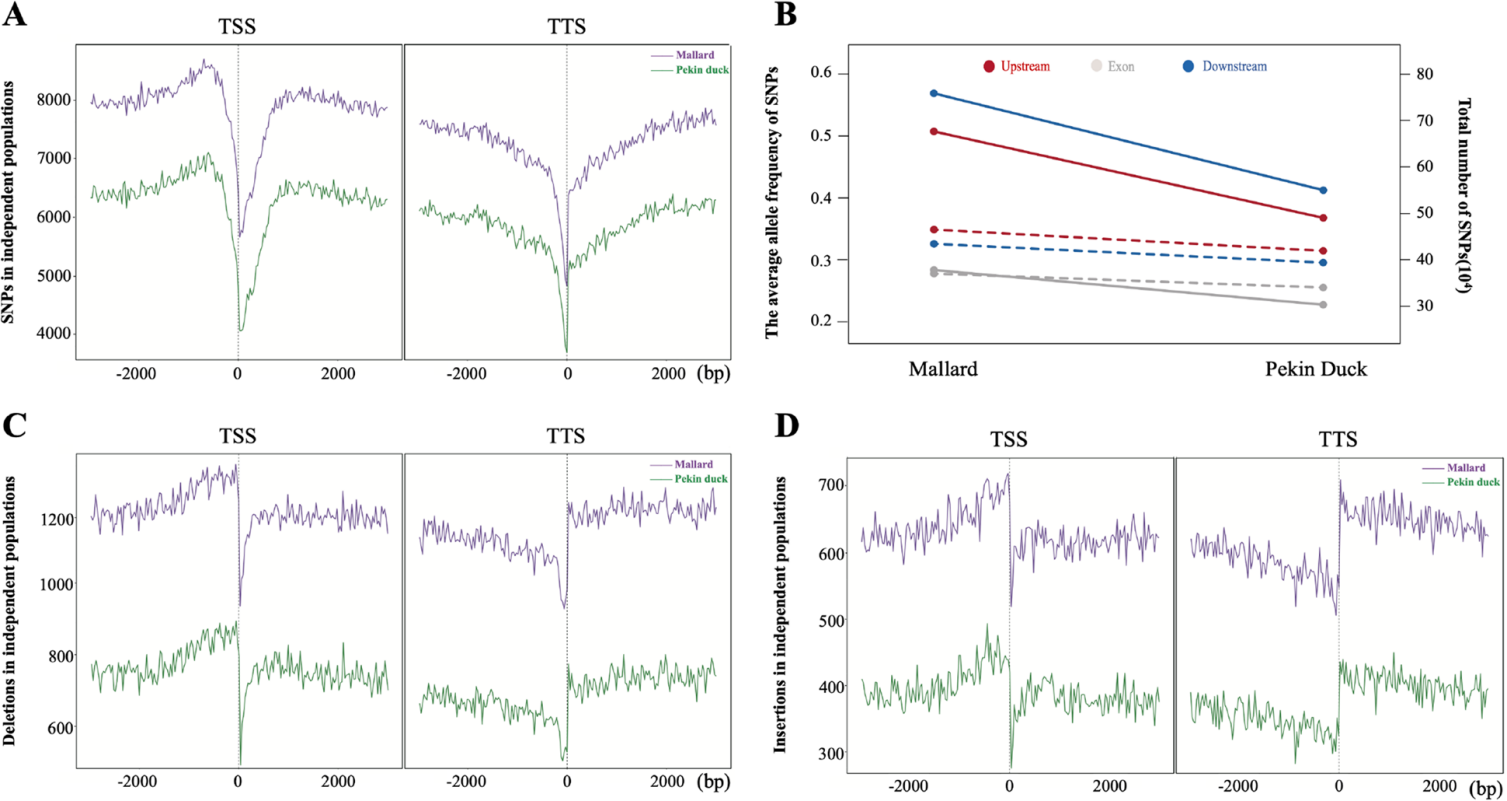
Characterization of variants during duck domestication. **A** The number of SNPs in the gene transcription start site (TSS) and transcription termination site (TTS) observed in ancestoral (mallard) and domesticated (Pekin) duck populations. The number of SNPs per 30 bases represents the sum of the gene model. **B** The *y*-axis on the left and right represents the average allele frequency of SNPs and the total number of SNPs detected in different gene structures in the mallard and Pekin duck populations. The solid line represents the total number of SNPs, and the dashed line represents the average allele frequency of SNPs. **C** The number of deletions in the TSS and TTS observed in mallard and Pekin duck populations according to the gene model. **D** The number of insertions in the TSS and TTS observed in mallard and Pekin duck populations according to the gene model

The genome resequencing analysis from 40 mallard samples provided 14,716,112 high-quality single-nucleotide polymorphisms (SNPs). Of these, 678,135 SNPs were in upstream regions (~ 2 kb), 570,453 SNPs were downstream of a gene (~ 2 kb), and 286,395 SNPs were within exons. The 45 Pekin ducks generated 10,268,646 high-quality SNPs, with 488,349 upstream SNPs, 411,772 downstream SNPs, and 213,624 exonic SNPs (Table S2). Further, we annotated the variants using variant effect predictor (VEP) and found the distribution of SNPs, and InDels around the genes was obviously lower in the transcription start site (TSS) or transcription terminator site (TTS) of genes (Fig. 1A). Compared with mallard genomes, the number of SNPs in the upstream region of protein-coding genes in Pekin duck decreased by 27.99% (from 678,135 to 488,349), and the average allele frequency increased from 0.266 in the mallard population to 0.351 in the Pekin duck population (Fig. 1B). Similarly, the number of InDels in Pekin duck genomes (4,449,192) was 32% less than in mallards (6,529,322), and the number of InDels in upstream and downstream regions was higher than that in the gene body (exons and introns) regions (Fig. 1C and D). Initial analysis showed that domestication has potentially decreased the diversity in both gene flanking and gene body regions in Pekin ducks.

### Characteristics of genes under selection during duck domestication

**Fig. 2:**
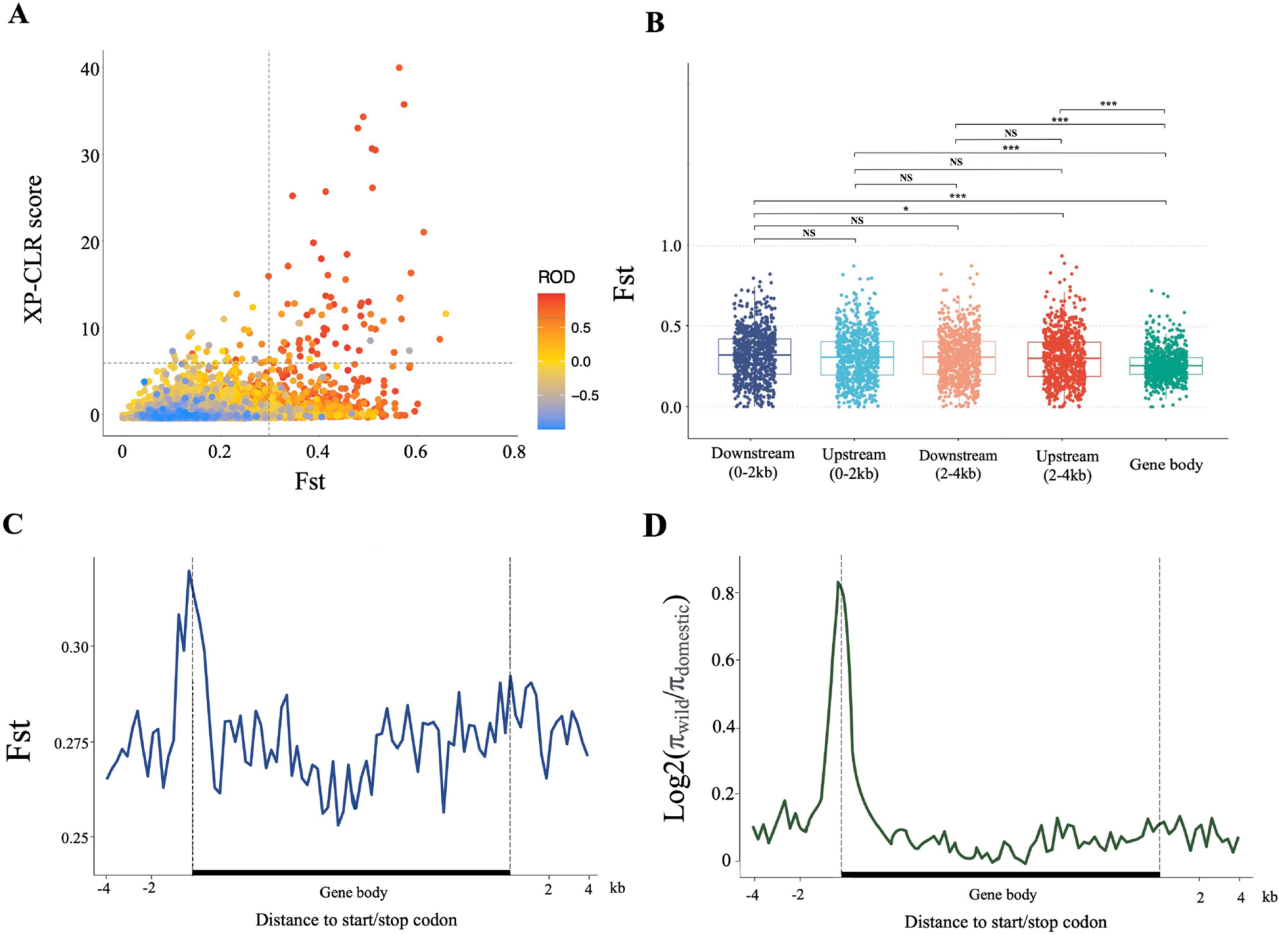
Characterization of selection forces in different structures of genes. **A** Distribution of the pairwise fixation index (F_ST_) (*x*-axis), XP-CLR score (*y*-axis), and value of ROD (color) between wild-type and domesticated ducks. The dashed vertical and horizontal lines indicate the significance threshold (*F*_ST_ > 0.31, *ROD* > 0.668, *XP-CLR* > 6.685) used for extracting outliers. **B** Difference test of F_ST_ in different structures of 781 protein-coding genes in genome selected regions. The level of significance is presented as ns (not significant), *(*P* < 0.05), or ***(*P* < 0.001). **C** Distribution patterns of F_ST_ in different structures of gene model, including upstream 4-kb region, gene body, and downstream 4-kb region. The blue solid line represents the mean value of F_ST_ for 781 putative selected genes, and the dashed line represents the gene transcription start site and transcription termination site. The gene body region on the *x*-axis does not represent the actual sequence length; each gene is scaled according to the sequence length. **D** The distribution of the degree of nucleotide diversity loss in mallard and domesticated duck populations was calculated using the same genetic model of Fig. 2**C**

We combined mallard and Pekin duck populations for variant detection, generating a final set of 12,609,352 SNPs to identify genomic regions and genes under selection during domestication after filtering. To improve the accuracy of detecting the selected regions, we used three independent methods to identify the selection signals of domestication in the duck genome. We compared the Pekin duck population with mallards by estimating pairwise genetic differentiation (F_ST_), reduction of diversity (ROD), and cross-population composite likelihood ratio test (XP-CLR) in 40-kb sliding windows along the genome. We defined the top 5% ranked windows in at least two statistics as putative selective sweep regions (*F*_ST_ > 0.31, *ROD* > 0.668, *XP-CLR* > 6.685). After merging consecutive outlier windows, 322 regions containing 781 protein-coding genes were identified (Fig. 2A and Table S3).

Next, we compared the differentiation characteristics of different gene structures of these 781 selected genes. Differentiation signature screening tests showed that the F_ST_ in the upstream and downstream regions of these genes was significantly higher than that in the gene body (*P* < 2.2e-16), indicating that the gene body was under stronger purifying selection (Fig. 2B). Although the F_ST_ between regions which were 0-2 kb and 2-4 kb upstream and downstream of genes were not significantly different, we can clearly observe that the region closer to the gene transcription start site (TSS) has higher F_ST_, and the region with highest F_ST_ is near the TSS (Fig. 2C). This phenomenon was not observed in downstream regions, which had F_ST_ values almost as evenly distributed as the gene bodies. The calculation of nucleotide polymorphisms found that the closer to the gene body in the upstream region, the more nucleotide polymorphisms were lost in the domestic ducks, while the distribution of nucleotide polymorphism in the downstream region was as random as in the gene bodies (Fig. 2D). For the gene body, the proportion of different variant types between mallard and Pekin duck populations is basically the same as that of the whole protein-coding gene sets, with no significant difference in high effect variants, such as splicing variants and missense mutation, indicating the selected features of upstream regions were more obvious than that of gene body regions (Table S4). Importantly, we used the same method to detect the F_ST_ and nucleotide polymorphisms in the upstream region of the selected genes in another meat-type Pekin duck (maple leaf duck) and three egg-laying and dual-purpose type ducks (Shaoxing duck, Gaoyou duck, and Jinding duck). The results showed that, for ducks, the differentiation features of the upstream region close to the gene TSS were the most significant during domestication (Fig. S1).

To clearly understand the role of selection on upstream regions in phenotypic changes occurring during duck domestication, we wanted to identify the genes showing an upstream selection from the genome-wide selected genes. We ranked the F_ST_ of the upstream region of the whole-genome protein-coding gene set and identified 304 genes with selected promoters (*F*_ST_ > 0.299, 5%; Table S5). For fixed SNPs (frequency of SNPs = 1 or 0), 5.3% of SNPs in the Pekin duck and 0.7% in the mallard were fixed in the upstream region of upstream selected genes, with the average number of fixed SNPs being 0.2% in the Pekin duck and 0.09% in mallard (based on whole-genome variants). Thus, for the Pekin duck, the proportion of fixed SNPs in upstream selected genes is 25 times greater than the proportion for randomly selected genomic loci. In addition, simulation analyses were conducted to test whether or not the observed differences in diversity patterns can be explained solely as an artifact of demographic bottlenecks during domestication. In addition, simulation analyses were conducted to test whether or not the observed differences in diversity patterns can be explained solely as an artifact of demographic bottlenecks during domestication. We simulated the fixation index between Pekin duck and mallard duck populations during domestication, and the results showed that the fixation index of the genes with selected promoters detected according to the actual data was caused by selection (Fig. S2). In summary, sequence diversity analysis indicated that there was strong differentiation in the upstream region of genes in the Pekin duck which occurred during the history of duck domestication.

### Analysis of transcriptional regulatory regions and 3D genome structure in duck

**Fig. 3:**
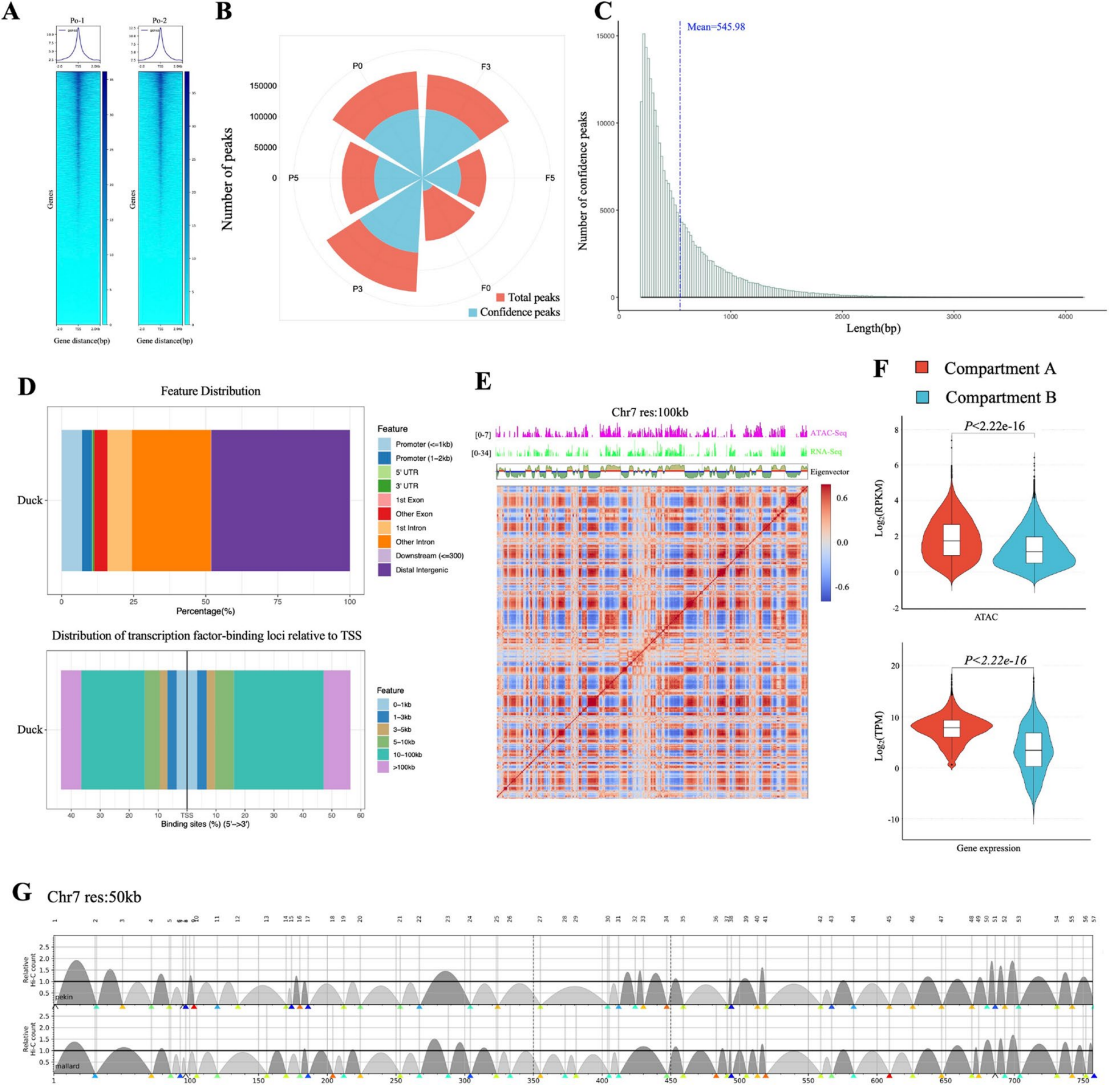
Potential regulatory regions and 3D structure of duck genome.** A** The ATAC-seq signal enrichment around the transcription start sites (TSSs) for two representative samples. **B** The number of narrow peaks detected in 12 samples and the narrow peaks detected by irreproducible discovery rate (IDR) was considered as confidence peaks (*P* < 0.01). The P0, P3, and P5 represent subcutaneous adipocytes that begin to differentiate and differentiate for 3 days and 5 days, respectively. The F0, F3, and F5 represent abdominal adipocytes that begin to differentiate and differentiate for 3 days and 5 days, respectively. **C** Length distribution of identified chromosomal accessibility regions. **D** The proportion and distance of chromatin accessibility regions annotated to the different structures of genes. The upper bar graph represents the proportion of chromatin accessibility regions annotated to the gene structure, such as promoter, exon, and UTR. The bar graph below shows the distance from the closest chromatin accessibility region to the gene TSS. **E** ATAC-seq and RNA-seq enrichment and correlation map of a Hi-C matrix for chromosome 7 at 100-kb resolution (res) in mallard breast muscle. The chromatin activity and expression level of the “A” compartment are higher than those of the “B” compartment. **F** Open chromatin and gene expression in the active “A” compartment (ATAC-seq *n* = 147,398; gene *n* = 8165) and the inactive “B” compartment (ATAC-seq *n* = 99,246; gene *n* = 3579). A two-sided unpaired Wilcoxon test was used to calculate *P*-values. **G** The TAD structures of chromosome 7 in mallard and Pekin ducks were detected at 50-kb resolution, among which mallard duck contained 48 TAD structures, and Pekin duck contained 43 TAD structures

We used 12 ATAC-seq datasets from adipocytes of Pekin duck to identify the open regions of duck chromatin that may contain different regulatory elements (Table S6). As expected, these chromatin-accessible regions were mainly located near the TSS of genes (Fig. 3A and Fig. S3). In total, 249,326 peaks were obtained with an average length of 546 bp after merging the confidence accessibility regions of all samples, accounting for about 11.24% of the whole genome (Fig. 3B and C). A total of 26,586 (10.66%) peaks were within 2-kb upstream of 5′-UTR regions, and the average distance to the TSS was 483 bp, providing accurate predictions of gene promoters for 14,435 (78.07% of all) protein-coding genes (Fig. 3D and Fig. S5). The prediction analysis of potential promoters showed that the upstream selected genes could be considered as genes with selected promoters.

The 3D structure of the genome is an important factor affecting gene transcription, regulation, and expression. The 3D structure of the duck genome was assessed using in situ Hi-C data where the skeletal muscle of mallard and Pekin duck was used as a representative tissue. In total, 3,725,771 and 4,430,634 paired-end reads were sequenced, yielding more than 100 × coverage of the duck genome. After filtering potentially artificial reads using the HiC-Pro pipeline, 18,043,966 and 18,379,643 unique valid interactions were obtained, among which 10,142,064 and 10,098,357 were cis-interactions in mallard and Pekin duck respectively (Table S7). We divided the genome into active “A” compartments (563.9 Mb, 46.53%) or the inactive “B” compartments (594.1 Mb, 49.02%) based on chromatin interaction frequencies and observed that the “A” compartments were highly enriched for actively transcribed genes and open chromatin signals (Fig. 3E and F) [28, 29]. In total, 218 genes with selected promoters were located in the “A/B” compartment, of which 157 (72.01%, compared with 79.77% at the whole-genome level) were located in “A” compartments and 61 (28.89%, compared with 20.23% at the whole-genome level) in “B” compartments, with more genes with selected promoters in “B” compartments than the whole-genome level (Table S5). At 50-kb resolution, 1206 and 1069 TADs were detected in wild (mallard) and domesticated (Pekin) ducks, with an average length of ~ 840 kb and ~ 956 kb, respectively, indicating that the TADs of mallard and Pekin duck genomes have changed during domestication (Fig. 3G). Only 244 TADs were predicted to be the same in wild and domesticated ducks, with 477 genes being near the boundary of differential TADs (around ~ 5 kb) (Table S8). These genes may bind to different regulatory factors and affect gene transcription levels in breast muscle in mallard and Pekin ducks [30, 31]. The duck epigenetic maps of predicted regulatory regions and 3D genomic structure will provide a new perspective on the different interactions between cis-regulatory elements in mallard and Pekin duck.

### The effect of promoter selection on gene expression

**Fig. 4:**
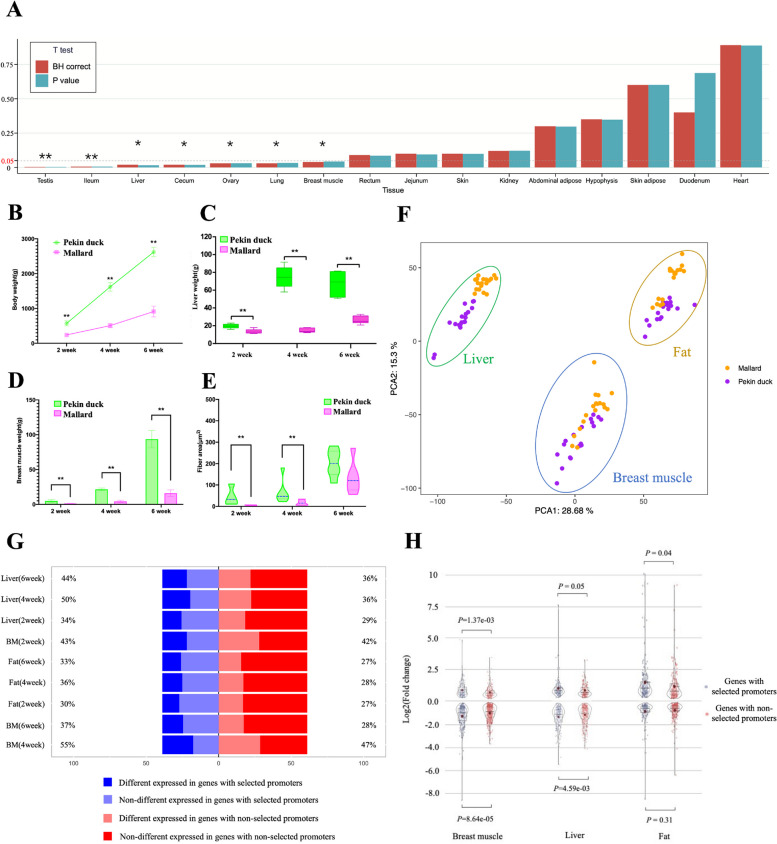
Gene expression characteristics of multiple tissues during duck domestication. **A** The differential test of genome-wide protein-coding genes from 16 important tissues between mallards and Pekin ducks. The differential expression test was done using *t*-test, and the level of significance is presented as ∗ (*P* < 0.05) and ∗  ∗ (*P* < 0.01). The comparison of body weight (**B**), liver weight (**C**), breast muscle weight (**D**), and muscle fiber area (**E**) between mallard and Pekin duck during dynamic development. The body weight and the breast muscle weight have increased significantly during duck domestication. **F** Principal variance component plots of the expression level of breast muscle, liver, and fat tissues in domesticated and wild duck samples. Each tissue contains six samples from three developmental stages (2 weeks, 4 weeks, and 6 weeks). The orange squares indicate wild-type ducks (mallard), and the purple squares indicate domesticated ducks (Pekin duck). PC, principal component. **G** The proportion of differentially expressed genes in genes with selected promoters (*n* = 304) and genes with nonselected promoter (*n* = 477) sets in different periods of breast muscle, the liver, and fat tissue. **H** The test of fold changes in whole differentially expressed genes in genes with selected promoters and genes with nonselected promoter sets. The up-regulated and down-regulated genes were tested by parameter test, and the *P*-value was corrected by Holm-Bonferroni method

For exploring gene expression differences in mallard and Pekin duck, we obtained RNA-seq data from 16 tissues including the testis, ileum, liver, cecum, ovary, lung, breast muscle, rectum, jejunum, skin, kidney, abdominal adipose, pituitary, skin adipose, duodenum, and heart (Table S1). The gene expression in the testis and ileum was very significantly different (*t*-test, *P* < 0.01) and in the liver, cecum, ovary, lung, and breast muscle was significantly different (*t*-test, *P* < 0.05) between Pekin duck and mallard, indicating that a wide range of gene expression changes relating to selected traits such as reproduction, growth, fat deposition, and feed conversion ratio occurred in the tissues studied during the course of domestication (Fig. 4A).

We recorded the phenotypes of mallard and Pekin duck in the early development stage (from 2 to 6 weeks after hatching). The body weight of the Pekin duck increased almost linearly during this developmental stage and reached three times the body weight of the mallard by 6 weeks (Fig. 4B). Similarly, the breast muscle and liver weight of the Pekin duck were significantly higher than that of mallard in the same period (Fig. 4C). The diameter and area of muscle fibers of Pekin duck were larger than those of mallard duck, as determined by H&E staining (Fig. 4D and E and Fig. S5). In addition, Pekin ducks have excellent fat deposition characteristics, with a fat percentage of more than 30% at 6 weeks, which is much higher than that of mallards in the same period [32]. Therefore, we selected breast muscle, the liver, and fat as representative tissues for dynamic transcriptome analysis to examine the expression patterns of genes with selected promoters in the selected trait-related tissues (Fig. 4F).

We identified the differentially expressed genes (DEGs) between mallard and Pekin ducks using DESeq2. There was an average of 7024, 6557, and 5121 (padj < 0.05) DEGs in breast muscle, liver, and fat tissue in each period, accounting for 37.99%, 35.46%, and 27.70% of all protein-coding genes respectively (Fig. S6). In genes with selected promoters, 267 genes (87.83%) were differentially expressed in domestic trait-related tissues (Table S5), and the average proportion of DEGs in breast muscle, liver, and fat tissue was 45%, 42.67%, and 33%, respectively, which were higher than the average proportion of DEGs in breast muscle (39%; Fisher’s exact test, *P* = 3.24e-04), liver (33.67%; Fisher’s exact test, *P* = 2.16e-12), and fat tissue (27.33%; Fisher’s exact test, *P* = 4.62e-3) for genes with nonselected promoters (Fig. 4G and Table S9). In addition, the average fold change of DEGs in breast muscle, liver, and fat tissues was 1.83, 1.69, and 1.71, respectively, which is lower than that of genes with selected promoters (2.17, 2.08, and 2.87, respectively) and genes with nonselected promoters (1.92, 1.77, and 2.71, respectively). Furthermore, in breast muscle, the fold change of both up-regulated (*P* = 1.37e-03) and down-regulated genes (*P* = 8.64e-05) in genes with selected promoters was higher than that in genes with nonselected promoters. The fold change of down-regulated (*P* = 4.59e-03) genes among genes with selected promoters in the liver and up-regulated (*P* = 0.04) genes among genes with selected promoters in fat was significantly higher than the equivalent in genes with nonselected promoters (Fig. 4H). The proportion and fold change of DEGs in genes with selected promoters were significantly different compared with the genes with nonselected promoters in breast, liver, and fat tissue.

Note that 46 genes with selected promoters that may relate to domestication traits were differentially expressed in at least one of the breast muscle, liver, and fat tissue, with 29 (59.18%) genes with selected promoters being associated with muscle weight, fat weight, and other economic traits in previous studies (Table S10), indicating that the expression of these genes with selected promoters may affect these traits. Among them, *BIN3*, which mainly has functions in protein localization and skeletal muscle fiber development [33], and control of myofiber size in vivo [34] had the highest F_ST_ value (*F*_ST_ = 0.83, *ROD* = 0.92) among promoters of genes with selected promoters related to muscle development. It is also differentially expressed in breast muscle between mallard and Pekin ducks. This gene was not only differentially expressed in breast muscle, but the expression was also higher in 16 different tissues of Pekin duck (domesticated) when compared to mallard (ancestral), indicating that the selection of regions upstream of genes has a broad effect on gene expression for genes with important function across multiple tissues (Fig. S7). In addition, *ELOVL3* showed the highest F_ST_ value in the upstream regions with the group of genes with selected promoters related to fat traits (*F*_ST_ = 0.66, *ROD* = 0.61). *ELOVL3* is known to be mainly involved in the synthesis of fat- and long-chain fatty acids [35, 36]. This gene also only showed significant differential expression in selected tissues related to the gene function, with the related level of expression being high (Fig. S8). These results indicate that the selection of gene promoters may be one of the factors affecting gene expression during domestication.

### Positive selection of *ELOVL3* promoter in Pekin duck

**Fig. 5:**
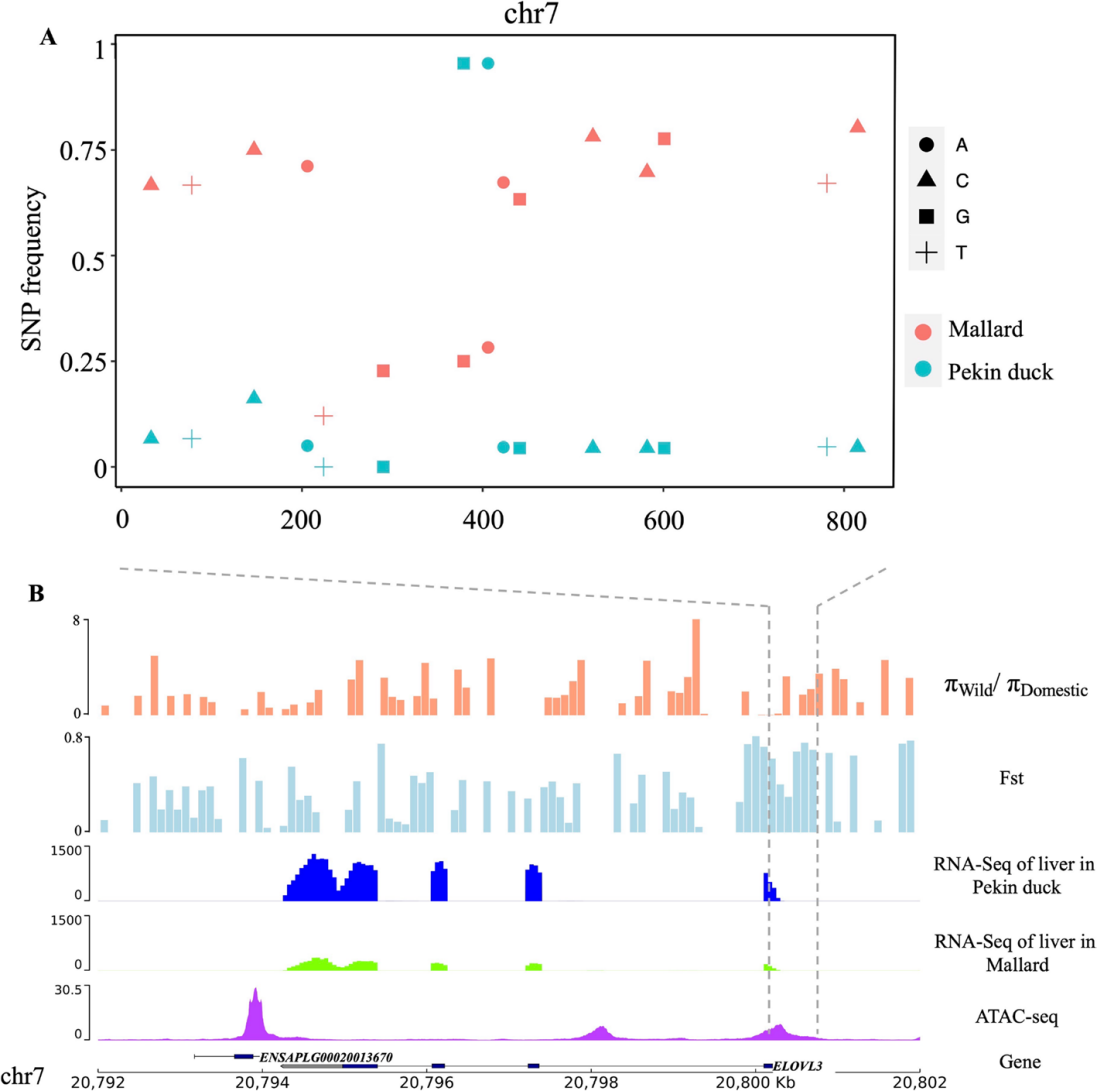
The multi-omics characteristics of the selected region which contains *ELOVL3 *in the mallard genome.** A** Distribution of SNP frequencies in the core promoter region of *ELOVL3* in wild and domestic duck populations. **B** Multi-omics signatures of the 10-kb genomic region which contains *ELOVL3*, including genomic selection signatures, gene expression, and chromosomal accessibility regions

*ELOVL3* is a key candidate gene with selected promoters for the increased fat content of Pekin duck. Here, we describe the genomic, epigenomic, and transcriptomic characterization of this region (chr7: 20,792–20,802 kb). The genome region surrounding *ELOVL3* (500 kb) contains a large number of specific favored variants, with the most strongly differentiated region near the TSS (*F*_ST_ = 0.66, *ROD* = 0.61), which includes potential regulatory elements (− 376~366 bp) (Fig. S9). The promoter region of *ELOVL3* contains 15 SNPs, of which 4 SNPs are completely fixed, and the frequency of 10 SNPs is 0.04 in Pekin duck, while the average frequency of these SNPs in mallard is 0.52, indicating that this region has been positively selected during domestication (Fig. 5A). As the main organ of fat synthesis, the liver shows the highest expression of *ELOVL3*, with the expression level in Pekin duck at 4 weeks after hatching being over four times greater than that in mallard (Fig. 5B).

Compared with the strong differentiation characteristics of the upstream regulatory region, there was weak differentiation (mean *F*_ST_ = 0.11) identified within the exons (except the first exon) of *ELOVL3* (Fig. 5B). In total, 54 SNPs were detected in the gene body of *ELOVL3*, including 46 intronic variants, 5 synonymous exonic variants, and 3 missense variants. The average frequencies of intronic (0.35 in mallards and 0.38 in Pekin ducks), synonymous (0.39 in mallards and 0.38 in Pekin ducks), and missense (0.03 in mallards and 0.02 in Pekin ducks) variants were similar in both mallards and Pekin ducks. In addition, we also examined the variant distribution of the core promoter region of *ELOVL3* in another meat-type Pekin duck (maple leaf duck) and three egg and dual-purpose type duck (Shaoxing duck, Gaoyou duck, and Jinding duck) populations (Fig. S11). The variant distribution pattern of the maple leaf duck and Shaoxing duck was similar to that of the Pekin duck, and the allele frequency of most variants was lower than mallard. These results suggest that these variants within the regulatory region may cause the expression changes of *ELOVL3* and subsequent increased fatty acid content in domesticated ducks.

### Variants in the *ELOVL3* upstream region affect fat content in duck

**Fig. 6:**
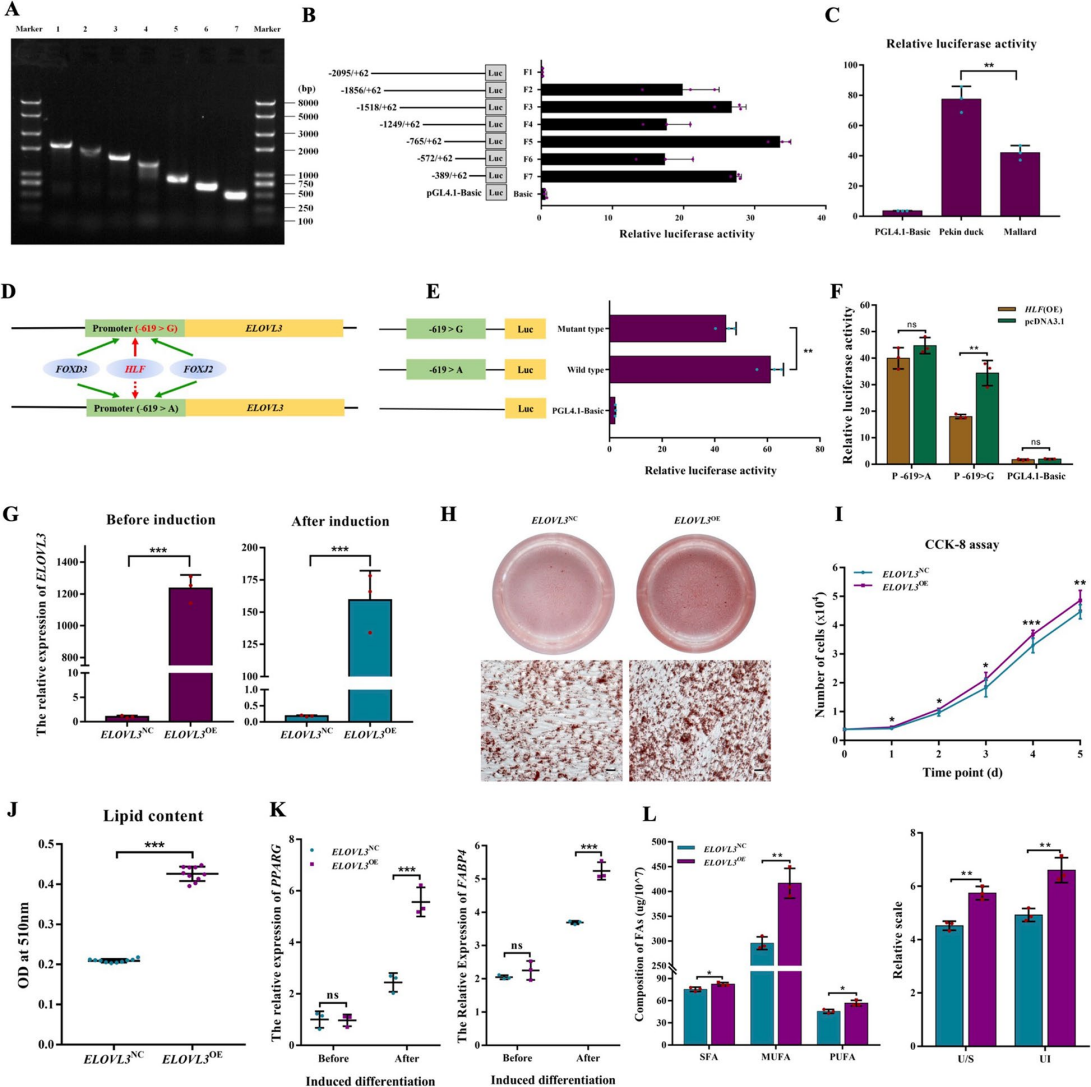
Effects of *ELOVL3* promoter region variant and expression on duck fat deposition and fatty acid composition. **A** Different lengths of a fragment of *ELOVL3* promoter region cloned by PCR. **B** Relative luciferase activity of different promoter fragments. F1–F7, fluorescence activity values detected after transfection of ICP1 cells with fluorescence vectors containing promoter truncated fragments of different lengths, using pRL-TK as reference (*n* = 3 biological replicates). **C** Relative luciferase activity of the mallard and Pekin duck core promoter haplotype vectors (*n* = 3 biological replicates). **D** Schematic diagram of transcription factor prediction which was affected by candidate causal SNP in core promoter regions. The solid line in the figure indicates the normal binding of transcription factors, and the dotted line indicates the reduced binding ability of transcription factors in the prediction results. The green line indicates the transcription factors that have not changed their binding ability before and after the mutation, and the red line indicates the transcription factors that have changed their binding ability after the mutation. **E** The relative luciferase activity of the mutant-type (− 619 > G-) haplotype and the wild-type (− 619 > A-) core promoter haplotype vectors in the ICP1 cell line (*n* = 3 biological replicates). Three luciferase reporter gene constructs were generated. They share identical backbone sequences except for the polymorphisms shown on the left. **F** The effect of transcription factor *HLF* on the relative luciferase activity (*n* = 3 biological replicates). **G** mRNA levels of *ELOVL3* were analyzed by Q-PCR in *ELOVL3*^*OE*^ and *ELOVL3*^*NC*^ cells before and after induction (*n* = 3 biological replicates). **H** Oil Red O staining to assess lipid accumulation at day 2 post-induction for *ELOVL3*^*NC*^ and *ELOVL3*^*OE*^ cells. The scale bar represents 20 μm (*n* = 3 biological replicates). **I** Cell Counting Kit-8 assay (CCK8) examines the proliferation of *ELOVL3*^*OE*^ and *ELOVL3*^*NC*^ cells over 5 days. Each cell number is counted by the standard curve established by CCK8 of the respective cells (*n* = 6 biological replicates). **J** The lipid droplet content of *ELOVL3*^*OE*^ and *ELOVL3*^*NC*^ cells obtained by Oil Red O staining and extraction methods (*n* = 3 biological replicates). **K** mRNA levels of PPARγ and FABP4 were analyzed by Q-PCR before and post-induction (*n* = 3 biological replicates). **L** Gas chromatography to assess fatty acid composition at day 2 post-induction for *ELOVL3*^*NC*^ and *ELOVL3*^*OE*^ cells. Data are shown as mean ± SD of three biological replicates. An independent sample *t*-test was used to analyze the statistical differences between groups. The level of significance is presented as ns (not significant), ∗ (*P* < 0.05), ∗  ∗ (*P* < 0.01), ∗  ∗  ∗ (*P* < 0.001)

To demonstrate the role of accumulated variants in the upstream region of *ELOVL3*, we further characterized the effect of *ELOVL3* core promoter mutations on gene expression levels and related phenotypic changes. Based on Pekin duck genomic DNA, we designed primers in the proposed promoter region of *ELOVL3* and amplified seven deletion fragments of different lengths: 2157 bp, 1918 bp, 1580 bp, 1311 bp, 827 bp, 634 bp, and 451 bp (Fig. 6A). To explore the active region of the *ELOVL3* promoter, the seven truncated fragments of the *ELOVL3* promoter were amplified and inserted into the pGL4.10 vector using restriction enzymes *Kpn I* and *Xho I* and were co-transfected into ICP1 cells with the pRL-TK vector for luciferase activity detection. The results showed that the core promoter region of *ELOVL3* is at − 765 ~  + 62 bp, and the − 389 ~  − 572bp region might have an element that negatively regulates promoter activity, while the − 572 ~  − 765bp region might contain a positive regulatory element (Fig. 6B). We constructed the haplotypes of the core promoter region of both mallard and Pekin duck and tested their transcriptional activity differences. The results showed that the promoter activity of the *ELOVL3* core promoter region from Pekin duck is significantly higher compared to that of mallard, and that this difference may affect gene expression levels (Fig. 6C). We predicted the binding of transcription factors affected by 15 SNPs in the core promoter region of *ELOVL3* and found that only the A to G mutation at the − 619 site affects binding of the *HLF* transcription factor, with the mutation significantly inhibiting the activity of the core promoter (Fig. 6D and E and Fig. S11). We then explored the regulatory effect of *HLF* on *ELOVL3* expression. Through co-transfection of the mutant plasmid and *HLF*-overexpression vector, we found that *HLF* can inhibit the activity of the core promoter region, especially at the − 619 sites, with the inhibitory effect of the G allele being extremely significant (Fig. 6F). Therefore, we conclude that continuous selection of the *ELOVL3* promoter region in Pekin duck has reduced the binding ability of *HLF*, thereby increasing the expression level of *ELOVL3*.

In mammals, *ELOVL3* is associated with fatty acid elongation, especially for very long-chain fatty acid biosynthesis [35, 36], but its function in avian fat deposition and fatty acid composition has not been fully resolved. Here, we compared the differences in proliferation and differentiation potential of ICP1 cells that overexpressed *ELOVL3* by transfection with plasmids carrying complete coding sequences (Fig. S12), with the empty plasmid as control. The expression level of *ELOVL3* in the overexpressed cell line was significantly higher compared to the control group before and after induction differentiation (*P* < 0.001; Fig. 6G). The deposition of triglyceride is a sign of adipocyte differentiation. We stained the neutral triglyceride in cells with Oil Red O stain to observe the difference in lipid deposition in different cell lines. The results show that cells overexpressing *ELOVL3* exhibited stronger adipogenic ability (Fig. 6H). In addition, we also added and tested further indicators that characterize the lipogenic capacity of cells, including the proliferation and differentiation of preadipocytes. The results showed that overexpression of the *ELOVL3* gene effectively promoted the process of avian adipogenesis, such as a > 20% increase in cell proliferation rate (Fig. 6I and Fig. S13), a more than 50% increase in lipid deposition content (Fig. 6J), and an increased expression of adipocyte marker genes (Fig. 6K). Importantly, we also measured the difference in the content and composition of various fatty acids in differently treated cells by gas mass spectrometry, and the results showed that overexpression of *ELOVL3* significantly increased the degree of unsaturation of fatty acids, among which the monounsaturated fatty acid (MUFA) and polyunsaturated fatty acid (PUFA) constituents were increased by 39% and 24%, respectively (Fig. 6L, Fig. S14 to 16, and Table S12). Furthermore, we sequenced the transcriptome of ICP cells before and after overexpression of *ELOVL3*. Compared with wild-type cells, 831 differentially expressed genes were specific to the induced differentiation after overexpression of *ELOVL3* (Table S13). Functional enrichment analysis showed that the overexpression of *ELOVL3* affected the expression of 59 genes related to fat and fatty acid synthesis (Fig. S17). These results suggest that the upstream regulatory regions of *ELOVL3* in Pekin duck were strongly differentiated and also play an important role in the improvement of fat content and fatty acid composition associated with domestication.

## Discussion

In this study, we have, for the first time, constructed a multi-omics map of mallard and Pekin duck, including phenotypic, genomic, transcriptomic, and epigenomic data, to explore the effect of gene promoter selection on gene expression and phenotype. This study provides a case model for understanding the profound impact of gene promoter selection on complex quantitative traits. From a phenotype perspective, domestication has significantly improved the growth rate, breast muscle weight, and fat content in Pekin duck compared with the ancestoral mallard. In selected genes identified in population genome resequencing data, the F_ST_ and loss of nucleotide polymorphisms in the upstream regions were higher than that in other gene regions. We also showed, through analysis of epigenome data, that the peak region of F_ST_ and loss of nucleotide polymorphisms are consistent with the predicted gene promoter region. Previously, we have observed that a high proportion (98.6%, 26,128/26,494) of significantly associated SNPs were located in regulatory or noncoding regions, and that the variation rate in these regulatory regions was much higher than in coding locations in the duck genome [26]. It has also been observed that variants with high allelic frequency differences are enriched in promoter regions compared to coding regions during dog domestication, indicating the importance of regulating regional variants in the process of domestication [37]. Here, we demonstrate that the selection of gene promoters was an important genetic mechanism for phenotypic variation during the duck domestication process and speculate that this phenomenon will be mirrored in other domesticated animals.

Many complex traits are affected by multiple minor alleles. One major source of variants is found within regulatory regions. Promoters have crucial roles in regulating gene expression [38, 39]. The continuous selection of favored variants in the promoter region could lead to the rapid fixation of SNPs in the promoter region. With modern breeding approaches, Pekin duck will fix favorable variants faster under high-intensity artificial selection. In our previous GWAS analysis in a large Pekin duck population, we found that the variants in the promoter regions of *TMEM74*, *ARSA*, *OTOGL*, *SNAP47*, and *CD164* genes were significantly associated with bone length, skin thickness, breast muscle toughness, daily feed intake, and the number of meals per day and had extreme allelic frequencies [32, 40–42]. We have observed that a large number of genes related to economic traits have undergone significant expression changes in domestic ducks [32, 41–46]. The significant differences in gene expression in the testis, ileum, liver, cecum, ovary, lung, and breast muscle between mallard and Pekin duck are consistent with the tissues expected to be important during the recent artificial selection associated with domestication. These differentially expressed genes with selected promoters are mostly connected with artificially selected traits, i.e., breast muscle weight and fat deposition, which also indicates that gene promoter selection plays an important role in regulating gene expression.

Pekin duck has a well-known ability for fat deposition compared to its wild ancestor and other domesticated ducks. Fat-related traits have been extensively selected in domesticated Peking ducks, as well as in some local duck breeds. There are multiple genes in the fat deposition pathway that have been selected, especially in the promoter region (Table S7). We explored several lines of evidence (F_ST_, ROD, XP-CLR, and selection simulation analysis) to confirm selection within the promoter of *ELOVL3*. We then linked the selected *ELOVL3* promoter region in Pekin duck with fat deposition and unsaturated fatty acid composition through a series of functional experiments. Previous studies have found that the rate of accumulation of saturated fatty acids (SFAs) (mainly C16:0) and MUFAs (mainly C16:1n-7 and C18:1n-9) in Pekin duck exceeds that of mallard, with the MUFA in Pekin duck being > 20% higher than that of mallard [47]. We found that the selected SNPs in the *ELOVL3* core promoter could alter binding to the *HLF* transcription factor and decrease transcriptional activity. Cell proliferation rate, lipid deposition content, and unsaturated fatty acid percentages were increased significantly in the *ELOVL3* overexpression cell lines, which was consistent with the previous studies demonstrating that *ELOVL3* is an important regulator of endogenous synthesis of very long chain fatty acids and triglyceride formation in fat tissue [35, 36]. We have focused on *ELOVL3* as an example to illustrate the important effect of promoter region selection on complex quantitative traits in duck domestication. Our research thus facilitates a deeper understanding of the genetic mechanism of quantitative trait changes during domestication and reveals that deeper understanding of promoter region variant selection will provide valuable resources for the improvement of economic traits in ducks as well as other domesticated animals.

## Conclusions

We characterized the variant accumulation and differentiation features for gene promoter regions during domestication using multi-omics data from mallard and Peking ducks, suggesting that selection and variant accumulation in gene promoter regions have played an important role in the regulation of phenotypic changes during duck domestication. Furthermore, as an example of an ideal candidate gene whose promoter region was selected during duck domestication, we used functional experiments to validate the multiple regulatory effects of the *ELOVL3* gene and a key variant in its promoter region on fat deposition content and fatty acid content.

## Methods

### Multi-tissues sample collection

We selected mallard as a wild-type duck and Pekin duck as a high-intensity domesticated duck. The blood, pituitary, abdominal adipose, skin adipose, breast muscle, ileum, jejunum, cecum, duodenum, rectum, kidney, ovary, liver, heart, testis, lung, and skin samples in Pekin duck were previously collected from 6-week-old animals [48], while these tissues in mallard were collected as part of this study. Blood was stored in anticoagulant tubes at − 20 °C. The tissue samples were quickly frozen in liquid nitrogen after collection for temporary storage, and then all samples were stored at − 80 °C. All experiments with ducks were performed under the guidance of ethical regulations from the Animal Care and Use Committee of China Agricultural University, Beijing, China (permit number: SYXK 2007–0023).

### Whole-genome resequencing library construction and sequencing

The DNA was extracted from the blood of 45 Pekin ducks at 6 weeks of age. A minimum of 15μg DNA was used for library construction using the Illumina TruSeq DNA Sample Prep Kit (Illumina, CA, USA). DNA was isolated using the DNeasy Blood & Tissue Kit (QIAGEN, ON, Canada), and purified genomic DNA was mechanically disrupted using a Bioruptor (Diagenode Inc., NJ, USA) to generate approximately 300bp inserts. Purified libraries were quality controlled using StepOnePlus (Applied Biosystems, MA, USA) after amplification. Finally, paired-end sequencing data with a read length of 150 bp were generated using the NovaSeq 6000 platform (Illumina, CA, USA).

### RNA-seq library construction and sequencing

To explore the differences in gene expression and function that occurred during duck domestication, we used the RNeasy Plant Mini Kit (Qiagen, USA) to extract RNA from 16 different tissues and 4 different experimentally treated ICP1 cells. RNA quality was assessed using an Agilent Bioanalyzer 2100 (Agilent Technology, USA), and RNA samples with RNA integrity number (RIN) > 9.0 were used for cDNA library preparation. The cDNA libraries for Illumina sequencing were prepared using the SureSelect strand-specific RNA library kit (Agilent Technology, USA), according to the manufacturer’s instructions, and sequenced using an Illumina HiSeq 4000 sequencer (Illumina, San Diego, USA) to obtain paired-end reads with an average length of 150 bp and over 6-Gb sequencing reads for each sample.

### ATAC-seq library construction and sequencing

Duck adipose tissue (20 mg) was collected before and after differentiation and ground in liquid nitrogen; 800,000 nuclei were isolated from the ground tissue using a previously reported method [49]. The transposition reaction mixture (12.5μL TD buffer, 10μL ddH2O, and 2.5μL TDE [Illumina, FC-121–1030]) was then added to the isolated nuclei. The reaction system was incubated at 37 °C for 30 min and immediately purified using the QIAGEN MinElute PCR Purification Kit (QIAGEN, 28,006). Equimolar amounts of adapter 1 and adapter 2 were added after transposition, followed by PCR to amplify the library. After the PCR reaction, the library was purified using AMPure beads, and the library quality was assessed using Qubit, and finally, PE150 sequencing was performed using the Illumina HiSeq 4000 platform.

### Identification of variants occurring during the domestication of ducks

Raw reads were processed to remove the adapter and low-quality sequences using Trimmomatic [50]. The clean data from mallards and Pekin ducks were aligned to the mallard genome (Ensembl release: 107, CAU_wild 1.0) using bwa-mem [51] with the parameters: “bwa mem -M -R.” The standard process of SpeedSeq [52] was used to detect SNPs and small InDels. The raw variants were filtered using Vcftools [53] according to the following parameters: –maf 0.05 –max-maf 0.99 –minDP 3 –maxDP 60 –min-alleles 2 –max-alleles 2 –max-missing 0.1. SVs were detected by Delly [54], Manta [55], and LUMPY [56] (except for insertions). In order to improve the detection accuracy, SURVIVOR [57] was used to filter the results by keeping the variants detected by at least two methods with the following parameters: “SURVIVOR merge sample 1000 2 1 1 0 50.” Long segment variants between mallard and Pekin duck genomes such as the presence/absence variants (PAVs) have been reported in our previous study [26]. Finally, VEP [58] was used to annotate the functional effects of SNPs and small InDels with the parameters: “–distance = 2000.” The specific variants favored by selection in selective sweeps was captured with phased genotypes using iSAFE [59] with parameter –window 36 –step 18 –MaxRank 2 –MaxFreq 0.9. The annotated code for gene models and variant statistical analyses is available on GitHub (https://github.com/greymonroe/mutation_bias_analysis) [60].

### Genome scanning to detect selected regions

We used filtered SNPs to scan the selected regions for the entire genome. VCFtools [53] was used to calculate *F*_ST_ and *π* between the wild and domesticated populations, and the XP-CLR software was used to calculate the XP-CLR [61]. For the whole-genome scan, 40-kb windows were selected for the analysis of *F*_ST_, *π*, and XP-CLR, and the step of *F*_ST_ and XP-CLR was 10 kb. The reduction of diversity (ROD) value was calculated based on the ratio of *π* for a subpopulation with respect to a control subpopulation. The windows with more than 20 SNPs were reserved, and the top 5% windows, obtained by at least two methods, were regarded as the regions selected during domestication. For the selection pressure of gene structures, the *F*_ST_ and *π* were calculated with a window length of 1 kb, and only the windows containing the SNPs were kept for analysis.

### Preprocessing of the ATAC-seq datasets

The ATAC-seq data were processed (trimmed, aligned, filtered, and quality checked) using the ATAC-seq pipeline as previously described [62]. Read coverage of genomic regions of filtered BAM files was calculated by the multiBamSummary bins function in deepTools [63] to assess the genome-wide similarity of replicated BAM files with 100-bp bin size. Pearson correlation was calculated using plotCorrelation between repeated samples. Samples with a Pearson correlation coefficient > 0.80 were considered to have good repeatability and were used for subsequent analysis. The model-based analysis of ChIP-seq (MACS2) software [64] was used to identify the peak regions with options -g 1,210,757,325 –nomodel –shift -100 –extsize 200 -q 0.05. The irreproducible discovery rate (IDR) method was used to identify reproducible peaks between two technical replicates. Only peaks that were reproducible between two technical replicates were considered as confident peaks. All organized peaks were then combined into one standard peak list. The number of raw reads mapped to each standard peak was calculated using the cross function of BEDTools [65]. The raw count matrix was normalized by reads per kilobase of peaks, per million mapped reads (RPKM). The identified narrow peaks (*P* < 10^−5^) were annotated using ChIPseeker [66] and mallard genome annotation files (Ensembl, release 107).

### Preprocessing of the Hi-C datasets

The paired-end Hi-C reads from different libraries were mapped separately to the mallard genome (CAU_wild1.0) using the HiC-Pro pipeline [67] with the parameter “-s mapping.” The configuration file for Hi-C processing was prepared with MIN_MAPQ = 30, BIN_SIZE = 10,000, 20,000, 50,000, 100,000, 200,000; LIGATION_SITE = AAGCTAGCTT. Data normalization was then performed using TADbit [68] to generate contact matrices with 10-kb, 50-kb, 100-kb, and 200-kb resolutions. The A/B compartment was subjected to principal component analysis (PCA) at 100-kb resolution using TADbit segments to further reveal active (“A” compartment) and inactive (“B” compartment) chromatin regions in the genome. TAD structure was identified by the –only_tads parameter of the TADbit segment at a resolution of 50 kb, and the standardized matrix file was obtained by the iterative correction and eigenvector decomposition (ICE) method [69].

### Preprocessing of the transcriptome datasets

Raw data were filtered for low-quality and short reads, and after removing adapters, clean data were used for gene expression analysis. The clean data were aligned to the mallard genome (Ensembl, release 107, CAU_wild 1.0) using Hisat2 [70], and then the number of raw reads mapped to the protein-coding genes was counted using HTSeq [71] according to the gene annotation. The *t*-test method was used to analyze the differences in genome-wide normalized gene expression levels (FPKM) between mallard and Pekin duck in multiple tissues, and the false-positive rate was corrected by the Benjamini and Hochberg (BH) method [72]. Raw read numbers in breast muscle, liver, and adipose tissue of Pekin ducks and mallards at 2, 4, and 6 weeks after hatching were normalized by DESeq2 [73]. The samples with poor repeatability according to the sample-to-sample distance were eliminated, and six samples were used for differentially expressed gene analysis. Genes with *FDR* < 0.05 were considered to be differentially expressed.

### Functional annotation analyses

The gene symbols from the mallard genome were used for GO functional annotation on the target genes using the Metascape website (https://metascape.org/gp/index.html, v3.5.20230101) [74]. Finally, we classified the protein-coding genes according to their GO biological process terms.

### Forward simulations of fixation index during duck domestication

Selection and population bottleneck are the two major factors that affect the genetic variants between domestic animals and wild ancestors, so we, therefore, conducted forward simulations using SLiM3 software [75] to distinguish the effects on variant allele frequency between the two possibilities. According to the research of duck population genetics and history, the duck was domesticated about 2500 years ago [76]. We simulated the changes in fixation index (*F*_ST_) for the upstream region of genes with selected promoters (the upstream length of each gene is 2000 bp) between Pekin duck and mallard populations from the Pekin duck domestication to now. We simulated a 608-kb chromosome segment, which only contained noncoding regions and did not include exon regions, etc. We modeled beneficial (*s* = 0.05) and deleterious (*s* =  − 0.001) mutations in noncoding regions assuming beneficial and deleterious mutation fitness effects estimated by Guillaume Martin and Torgerson et al. [77, 78]. We assumed that 4% of noncoding mutations were deleterious [79], and 5% were beneficial mutations [78], and all other noncoding mutations were neutral. The sizes of the simulated initial mallard and Pekin duck population are 8433 and 4502, respectively, which are estimated using actual resequencing data based on the association between linkage disequilibrium and effective population size proposed by Sved J. A. et al. [80]. For all mutations, we assumed a mutation rate of 1.91e-9 mutations per site per generation following Nam et al. based on an estimate from the chicken genome [81]. In addition, we assumed a uniform recombination rate of 1e-8 crossovers per bp per generation, and each condition has 25 simulation replicates. For all simulations, we retained fixed mutations following the initial burn-in period, such that their impact on fitness was allowed to accumulate.

### Reverse transcription PCR (RT-PCR)

Total RNA extraction kit (e.z.n.a. a total RNA kit II reaction kit, Omega Bio-Tek, USA) was used to extract the RNA of the ICP1 cell line before and after induction of differentiation. After detection of RNA quality by 1% agarose gel electrophoresis and NanoDrop 1000 (Thermo Scientific, Wilmington, DE, USA), RNA was reverse transcribed using the PrimeScript™ RT reagent kit with gDNA Eraser reaction kit (Takara bio, USA) and used in quantitative PCR reactions with TB green premix Ex Taq™ fluorescence quantitative kit (Takara, USA). Q-PCR was performed using the ABI-7500 thermal cycler. Using the cDNA sequence obtained by reverse transcription as a template, the Primer-BLAST module of NCBI (www.ncbi.nlm.nih.gov/tools/primer-blast) was used to design gene-specific quantitative primers. The expression level of the target gene was normalized with *GAPDH* as the internal reference gene in each sample, and the relative expression level was calculated by the 2^−ΔΔCT^ relative quantification method [82]. Details of the primers used in the study are shown in Table S14.

### Truncation of the *ELOVL3* promoter region

Primers were designed using Primer3 (https://bioinfo.ut.ee/primer3-0.4.0/) for the 2500bp upstream sequence of the *ELOVL3* gene. The primer sequence information for the truncated region of the promoter is shown in Table S14. Amplification and purification of target fragments were done using the methods described above. The pGL4.10 restriction endonuclease site was analyzed using SnapGene 5.3.1, followed by double digestion and purification of the linearized vector using *Kpn* I and *Xho* I (NEB, USA). Finally, each fragment was constructed into a linearized pGL4.10 reporter vector using homologous recombination reagents.

### Cell culture, transfection, and dual-luciferase assay

A cell line of immortalized chicken preadipocytes (ICP1) was a kind gift from the Poultry Breeding Group of the College of Animal Science and Technology, Northeast Agricultural University, China [83]. ICP1 cells were cultured at 37 °C with 5% CO_2_ and 95% air humidity in DMEM/F12 medium with 10% fetal bovine serum and 1% penicillin and streptomycin. After 24 h from plating, transfection was carried out when the adherent coverage of cells reached about 80%, and pGL4.10 expression vector (0.475 μg), pRL-TK (0.025 μg), liposome (1.0 μL), and Opti-MEM of each cell were mixed and incubated for 10 min. After 6 h from transfection into ICP1 cells, medium was replaced, and cell samples were collected after 48 h. The cell lysate (100 µl) was centrifuged to obtain supernatant (20 µl) for use with a multifunctional enzyme marker (Infinite F200, CH) to measure the relative activity of firefly luciferase and Renilla luciferase (Dual-Luciferase Reporter Assay Kit, Vazyme bio, China).

### Construction of single-point mutation and overexpression vector

The original plasmid was used as a template, and the amplification primers with the single base mutation were introduced for reverse amplification. The information on the primer sequences is given in Table S15. The amplified product was digested with *DpnI* and then recombined and ligated after removing the methylated template plasmid. Finally, the product was transformed and cloned (Mut Express® II Fast Mutagenesis Kit V2, Vazyme bio, China). The CDS sequence of *HLF* in the duck was synthesized and constructed into the pcDNA3.1 overexpression vector.

### Construction of *ELOVL3*-overexpression cell lines

To construct the ch*ELOVL3*-overexpression vector, the full-length coding sequence of ch*ELOVL3* (Gene ID: 770,955; NCBI Reference Sequence: NM_001318410.2) was synthesized and cloned into the CMV promoter-driven piggyBac and an EF1α promoter-driven mCherry plasmid using NheI and SalI (New England Biolabs, Ipswich, MA, USA). The plasmid was a gift from Professor S. Wu (State Key Laboratory of Agrobiotechnology, College of Biological Sciences, China Agricultural University). The cloning plasmids were confirmed by sequencing. For *ELOVL3*^OE^ cell selection, ICPs were seeded in 6-well plates for further transfection using FuGENE® HD Transfection Reagent (Promega, Madison, WI, USA). After a 48-h recovery period, the cells were harvested using 0.25% trypsin/EDTA (Gibco, Gaithersburg, MD, USA) for identifying expression levels.

### ICP1 cell proliferation rate assay

The immortalized chicken preadipocyte cells (3800 per well) were cultivated in 96-well plates, and cell proliferation was detected after 5 days with the cell counting kit-8 (Dojindo, Kumamoto, Japan) at 450 nm using the Snergy™ HT Multi-Microplate Reader (Bio-Tek). All data were averaged from the results of six independent experiments.

### Detection of adipogenic differentiation of ICP1 cells

Adipogenic differentiation of ICP1 cells was induced using the previously described protocol [84]. ICP1 cells were expanded in culture using DMEM/F12 cell culture medium with 10% FBS. Cells at passages three to four were induced to differentiate after 2 days of confluence (day 0) with 160nM sodium oleate (Sigma-Aldrich, MO, USA) in DMEM/F12 supplemented with 10% FBS and 1% penicillin and streptomycin. After 2 days, cells were fixed with 10% formalin for 20 min and stained with Oil Red O (Beyotime-Bio, China) to examine lipid accumulation [85]. All experiments were repeated three times, and samples were treated in triplicate. Morphological changes were observed and photographed under an inverted fluorescent microscope (Nikon). Lipid droplet accumulation was measured by the Oil Red O extraction assay.

### Fatty acid composition determination and analysis

Dichloromethane-methanol solution (5 mL (2:1 v/v)) was added to 50 mg of each sample and put in a water bath at 80 °C. This was allowed to methylate for 30 min, and then 200 μL of the internal standard solution was added. This was followed by the addition of 1 mL of n-hexane extraction buffer. Reactions were then washed by adding 5 mL of pure water. Anhydrous sodium sulfate (100 mg) was added to 500-μL supernatant to remove excess water. The supernatant was mixed well and added to the injection bottle, ready for GC–MS detection, with the injection volume being 1 μL.

The samples were separated using an Agilent DB-23 column (60 m × 250 μm ID × 0.15 μm) gas chromatography system. The temperature program was as follows: the initial temperature was 50 °C, maintained for 3 min, heated to 220 °C at 10 °C/min, and held for 3 min, finally heated to 250 °C at 15 °C/min, and maintained for 10 min. The carrier gas was helium at a flow rate of 1.0 mL/min.

Mass spectrometry analysis was performed using an Agilent 7890–5977 gas-mass spectrometer. The inlet temperature was 280 °C, the ion source temperature was 230 °C, the transfer line temperature was 250 °C. Electron impact ionization (EI) source, SIM scanning mode, electron energy 70 eV.

The chromatographic peak areas and retention times were extracted by MassHunter software. A standard curve was determined, and the medium and long-chain fatty acid content of the sample was calculated (Table S16). Each set of samples contained six biological replicates.

To facilitate comparisons of FA composition, we calculated the unsaturation index (UI) and the unsaturated-to-saturated FA ratio (U/S) as reported by Wallaert and Babin [86]. The UI and U/S algorithm was as follows:

$$UI = \sum (\%monoenes + 2 \times \%dienes + 3 \times \%trienes)/100$$

$$U/S = \sum (\%UFA)/(\%SFA)$$

where monoenes, dienes, and trienes are fatty acids containing 1, 2, and 3 double bonds, respectively, and %, weight percentage; UFA, unsaturated fatty acids; and SFA, saturated fatty acids.

### Supplementary Information

**Additional file 1.** **Table S1.** Summary of multi-omics sequencing data during duck domestication. **Table S2**. Variation annotation detected in mallard and Pekin duck populations. **Table S3. **Genes that are located in the top 5% of selected regions. **Table S4.** The effect and number of variations in the gene body of selected genes in the mallard and Pekin duck population. **Table S5.** Selection and regulation characteristics of upstream selected genes. **Table S6.** Summary of ATAC-Seq data mapped to the mallard reference genome. **Table S7.** Summary of Hi-C data mapped to the mallard reference genome. **Table S8.** Genes located near the differential TAD boundaries between mallard and Pekin duck. **Table S9.** Different expression signatures of genes with selected promoters with potential regulatory regions in upstream of breast muscle, liver, and fat tissues. **Table S10.** The traits affected by genes with selected promoters with functions related to selected traits in other studies. **Table S11.** Quantitative Results of Medium and Long Chain Fatty Acids by Gas Mass Spectrometry. **Table S12.** Specific differentially expressed genes during adipocyte differentiation induced by overexpression of *ELOVL3* compared with wild type. **Table S13.** Sequence information relating to primers designed in this study. **Table S14.** Standard curve of Medium and Long Chain Fatty Acids by Gas Mass Spectrometry.

**Additional file 2: Fig. S1** The characteristics of FST and nucleotide polymorphism distribution of selected genes in MapleLeaf duck, ShaoXing duck, GaoYou duck, and JinDing duck. **Fig. S2** Simulation of fixation index between Pekin duck and mallard populations during duck domestication. **Fig. S3** The ATAC-seq signal enrichment around the transcription start sites (TSSs) for 10 representative samples. **Fig. S4** The distribution of the distance from peaks that were annotated to promoter region to the gene TSS. **Fig. S5** Comparison of breast muscle tissue and myofibers between mallard and Pekin duck during the rapid developmental stage after hatching. **Fig. S6 **The number of differentially expressed genes during dynamic development of breast muscle, liver, and fat tissue in mallard and Pekin duck. **Fig. S7** The expression profile of *BIN3 *in 16 tissues of mallard and Pekin duck. **Fig. S8** The expression profile of *ELOVL3 *in 16 tissues of mallard and Pekin duck. **Fig. S9** Selective sweep regions arising from domestication found around the *ELOVL3* region on chromosome 7. **Fig. S10 **The SNP sites and allelic frequencies of *ELOVL3 *core promoter region in local duck breeds. **Fig. S11** Construction of mutation at site -619 (A<G) in the upstream regulatory region of the *ELOVL3 *gene. **Fig. S12** Schematic diagram of duck *ELOVL3 *cDNA structure. **Fig. S13** CCK-8 Proliferation Assay Standard Curve for ICP1 Cells. **Fig. S14** Relative Standard Deviation (RSD) of within-batch QC samples. **Fig. S15** Metabolite Standard Mixture TIC Profile. **Fig. S16** Heatmap of biological replicates for fatty acid content determination by Gas Mass Spectrometry. **Fig. S17** The functional enrichment analysis to GO (Biology Process) of differentially expressed genes of ICP1 cell differentiation induced by overexpression of *ELOVL3*

## Data Availability

All data generated or analyzed during this study are included in this published article, its supplementary information files, and publicly available repositories, which we describe below. The whole-genome resequencing fastq files of Pekin duck and the ATAC-Seq data generated in this study are available at the National Center for Biotechnology Information (NCBI) with accession PRJNA878639 [87]. The Hi-C data of mallard and Pekin duck are available at NCBI with accession PRJNA554956 [88]. The RNA-Seq data are available at NCBI with accession PRJNA449259 [89], PRJNA878639 [87], and PRJNA645648 [90]. The whole-genome resequencing data are available at NCBI with accession PRJNA419832 [91], PRJNA450892 [92], and PRJNA896757 [93].

## Abbreviations

SNP: Single-nucleotide polymorphism
InDel: Insertions/deletion
SV: Structural variant
TAD: Topologically associating domain
VEP: Variant effect predictor
TSS: Transcription start site
TTS: Transcription terminator site
F_ST_: Fixation index
ROD: Reduction of diversity
XP-CLR: Cross-population composite likelihood ratio test
DEG: Differentially expressed genes
MUFA: Monounsaturated fatty acids
PUFA: Polyunsaturated fatty acids
SFA: Saturated fatty acids
