# Supplementary material for: Selection on the promoter regions plays an important role in complex traits during duck domestication
Source: BMC Biol. 2023 Dec 21;21:303. doi: 10.1186/s12915-023-01801-0 (PMC10740227; doi:10.1186/s12915-023-01801-0)
Supplement: Supplementary file 2 — Additional file 2: Fig. S1 The characteristics of FST and nucleotide polymorphism distribution of selected genes in MapleLeaf duck, ShaoXing duck, GaoYou duck, and JinDing duck. Fig. S2 Simulation of fixation index between Pekin duck and mallard populations during duck domestication. Fig. S3 The ATAC-seq signal enrichment around the transcription start sites (TSSs) for 10 representative samples. Fig. S4 The distribution of the distance from peaks that were annotated to promoter region to the gene TSS. Fig. S5 Comparison of breast muscle tissue and myofibers between mallard and Pekin duck during the rapid developmental stage after hatching. Fig. S6 The number of differentially expressed genes during dynamic development of breast muscle, liver, and fat tissue in mallard and Pekin duck. Fig. S7 The expression profile of BIN3 in 16 tissues of mallard and Pekin duck. Fig. S8 The expression profile of ELOVL3 in 16 tissues of mallard and Pekin duck. Fig. S9 Selective sweep regions arising from domestication found around the ELOVL3 region on chromosome 7. Fig. S10 The SNP sites and allelic frequencies of ELOVL3 core promoter region in local duck breeds. Fig. S11 Construction of mutation at site -619 (A<G) in the upstream regulatory region of the ELOVL3 gene. Fig. S12 Schematic diagram of duck ELOVL3 cDNA structure. Fig. S13 CCK-8 Proliferation Assay Standard Curve for ICP1 Cells. Fig. S14 Relative Standard Deviation (RSD) of within-batch QC samples. Fig. S15 Metabolite Standard Mixture TIC Profile. Fig. S16 Heatmap of biological replicates for fatty acid content determination by Gas Mass Spectrometry. Fig. S17 The functional enrichment analysis to GO (Biology Process) of differentially expressed genes of ICP1 cell differentiation induced by overexpression of ELOVL3 [file 12915_2023_1801_MOESM2_ESM.pdf]

Supplementary Materials for

Selection on the promoter regions play an important role for complex traits during duck domestication

Zhong-Tao Yin *et al.*

This PDF file includes:

Fig. S1. The characteristics of F_{ST} and nucleotide polymorphism distribution of selected genes in MapleLeaf duck, ShaoXing duck, GaoYou duck, and JinDing duck.
Fig. S2. Simulation of fixation index between Pekin duck and mallard populations during duck domestication.
Fig. S3. The ATAC-seq signal enrichment around the transcription start sites (TSSs) for 10 representative samples.
Fig. S4. The distribution of the distance from peaks that were annotated to promoter region to the gene TSS.
Fig. S5. Comparison of breast muscle tissue and myofibers between mallard and Pekin duck during the rapid developmental stage after hatching.
Fig. S6. The number of differentially expressed genes during dynamic development of breast muscle, liver, and fat tissue in mallard and Pekin duck.
Fig. S7. The expression profile of *BIN3* in 16 tissues of mallard and Pekin duck.
Fig. S8. The expression profile of *ELOVL3* in 16 tissues of mallard and Pekin duck.
Fig. S9. Selective sweep regions arising from domestication found around the *ELOVL3* region on chromosome 7.
Fig. S10. The SNP sites and allelic frequencies of *ELOVL3* core promoter region in local duck breeds.
Fig. S11. Construction of mutation at site -619 (A<G) in the upstream regulatory region of the *ELOVL3* gene.
Fig. S12. Schematic diagram of duck *ELOVL3* cDNA structure.
Fig. S13. CCK-8 Proliferation Assay Standard Curve for ICP1 Cells.
Fig. S14. Relative Standard Deviation (RSD) of within-batch QC samples.
Fig. S15. Metabolite Standard Mixture TIC Profile.
Fig. S16. Heatmap of biological replicates for fatty acid content determination by Gas Mass Spectrometry.
Fig. S17. The functional enrichment analysis to GO (Biology Process) of differentially expressed genes of ICP1 cell differentiation induced by overexpression of *ELOVL3*.

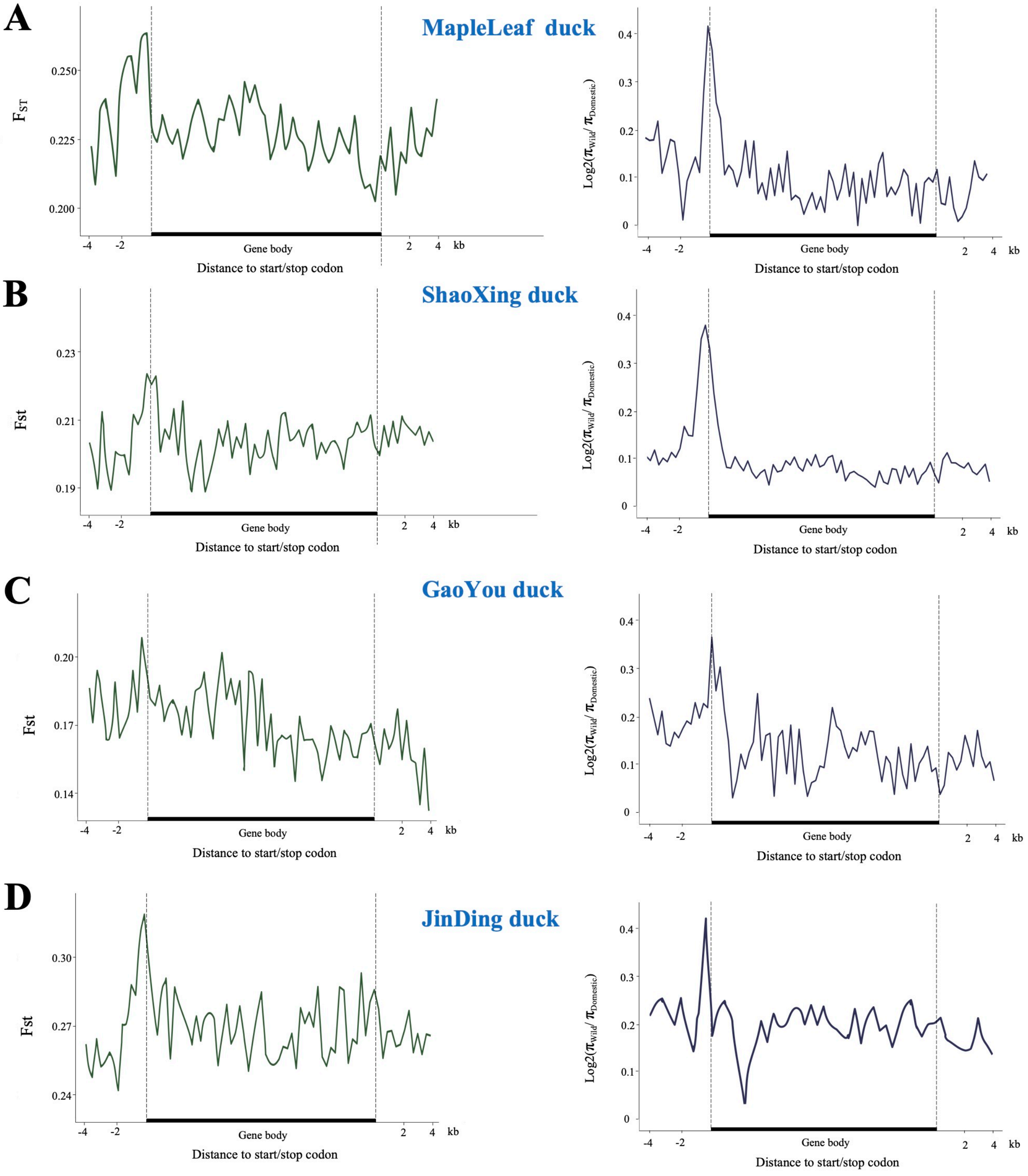

Fig. S1. The characteristics of F_{ST} and nucleotide polymorphisms distribution of selected genes in MapleLeaf duck (A), ShaoXing duck (B), GaoYou duck (C) and JinDing duck (D).

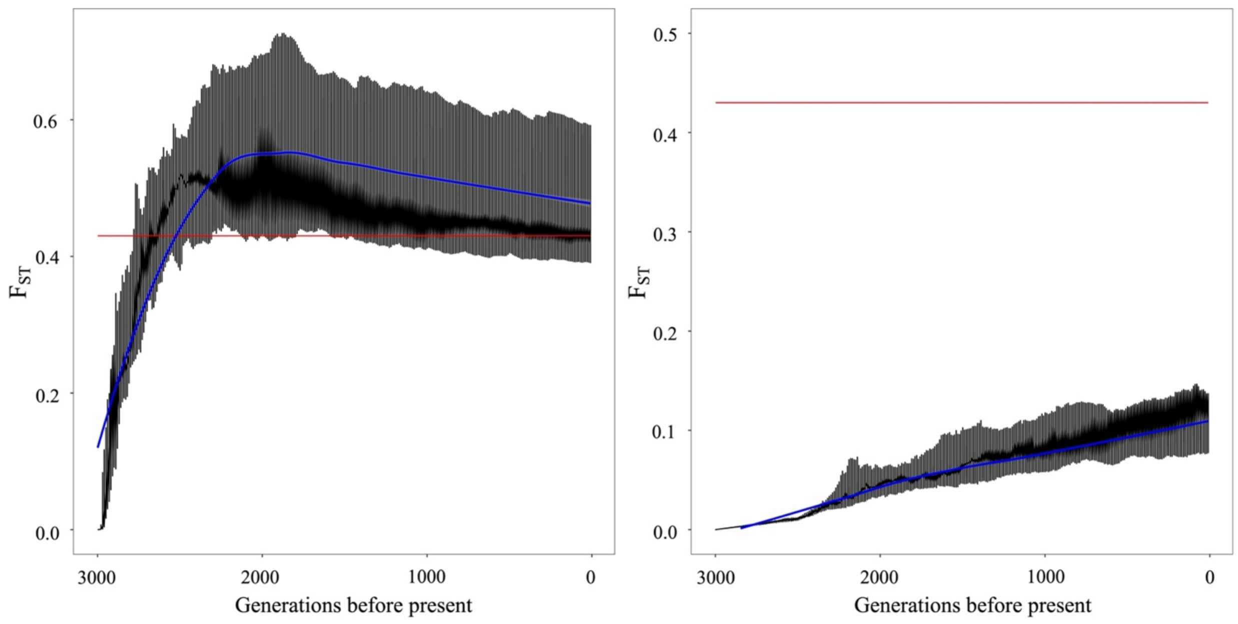

Fig. S2. Simulation of fixation index between Pekin duck and mallard populations during duck domestication. The left represents the simulation conditions with selection coefficients, and the right represents the simulation conditions with only bottleneck effects. The gray line represents the results of 25 simulation repeats, the blue line represents the results of 25 simulation fittings (95% confidence interval), and the red line is the fixation index we calculated using actual population resequencing data.

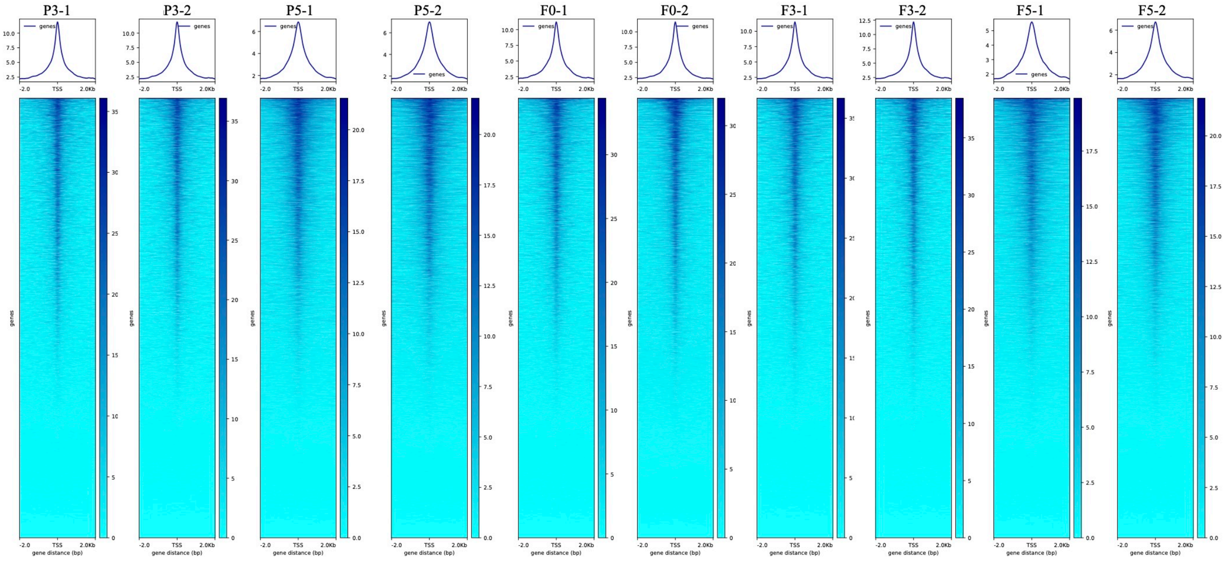

Fig. S3. The ATAC-seq signal enrichment around the transcription start sites (TSSs) for 10 representative samples (adipocytes of Pekin duck).

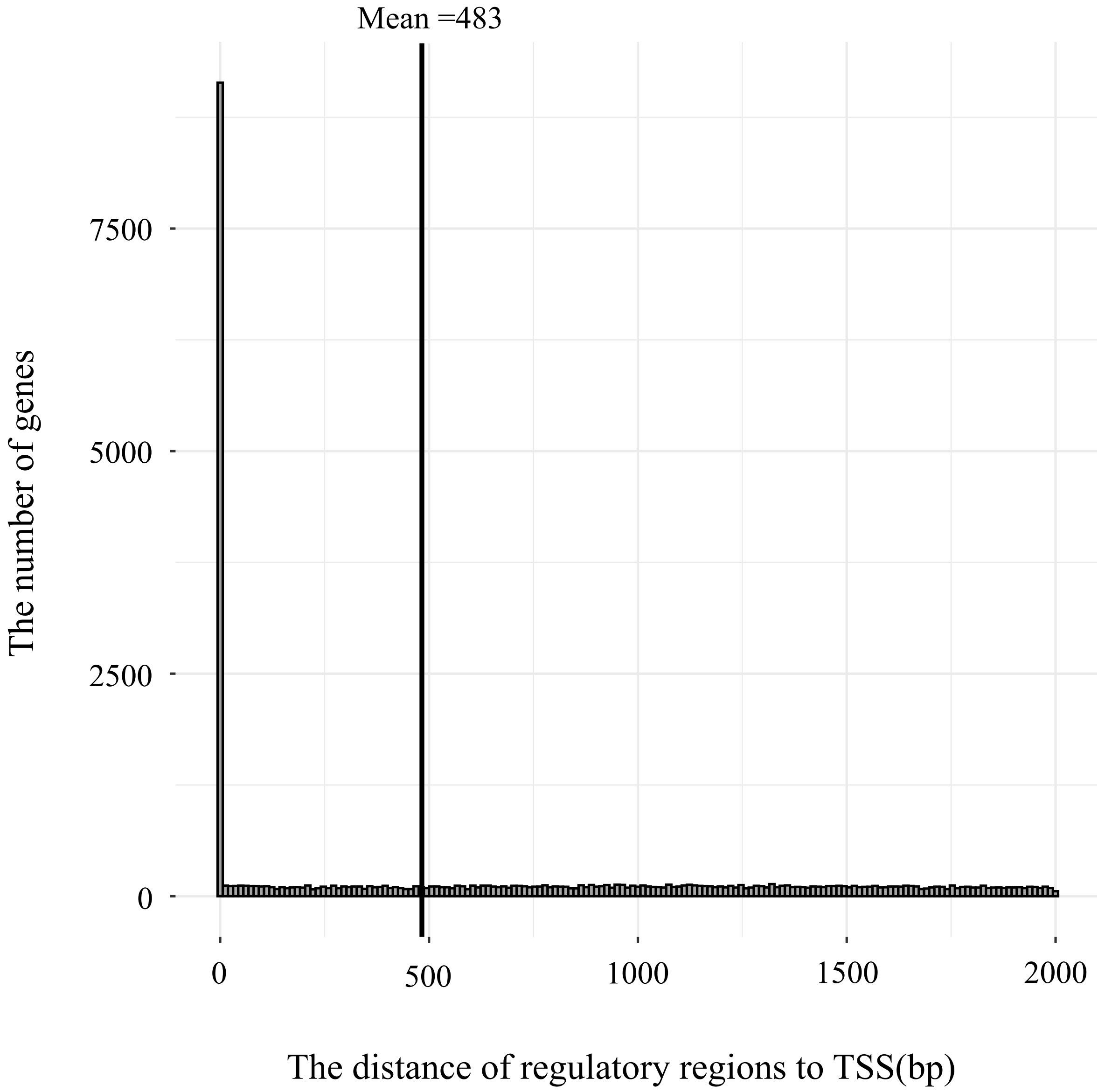

Fig. S4. The distribution of the distance from peaks which were annotated to promoter region to the gene TSS. As the figure shown, most of the genes containing the regulatory regions of the promoter were distributed near the gene TSS, and the average distance is 482bp.

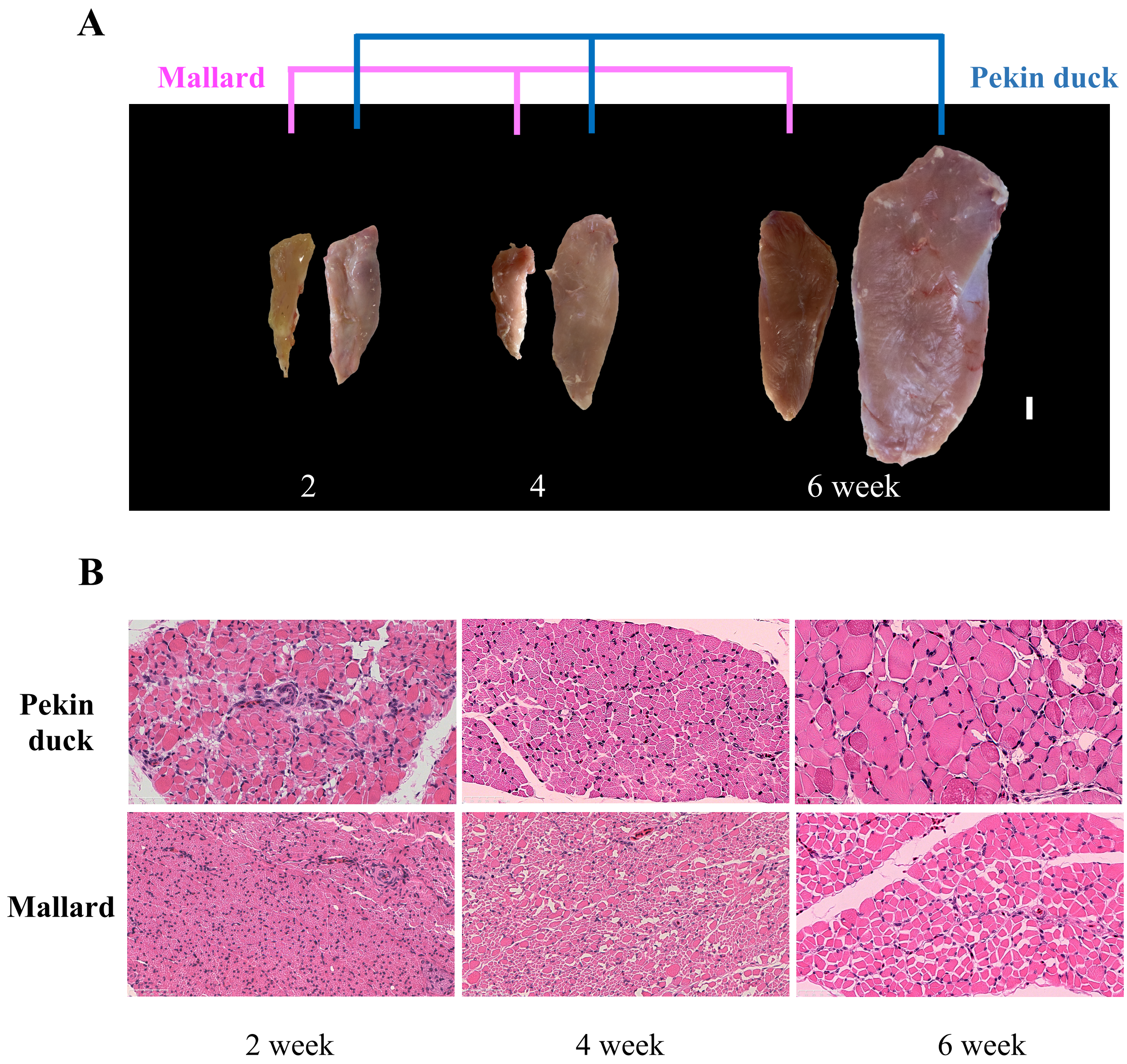

Fig. S5. **Comparison of breast muscle tissue and myofibers between mallard and Pekin duck during rapid development stage after hatching.** (A) phenotypes of breast muscle in mallard and Pekin duck. Numbers bottom the graphs are weeks. Scale bar, 1 cm. (B) H&E staining of breast muscle in mallard and Pekin duck at different developmental stages, showing the number and area of myofiber in Pekin duck is more than mallard. Scale bars, 25 μm in the figure. The violet point in figure is cell nucleus.

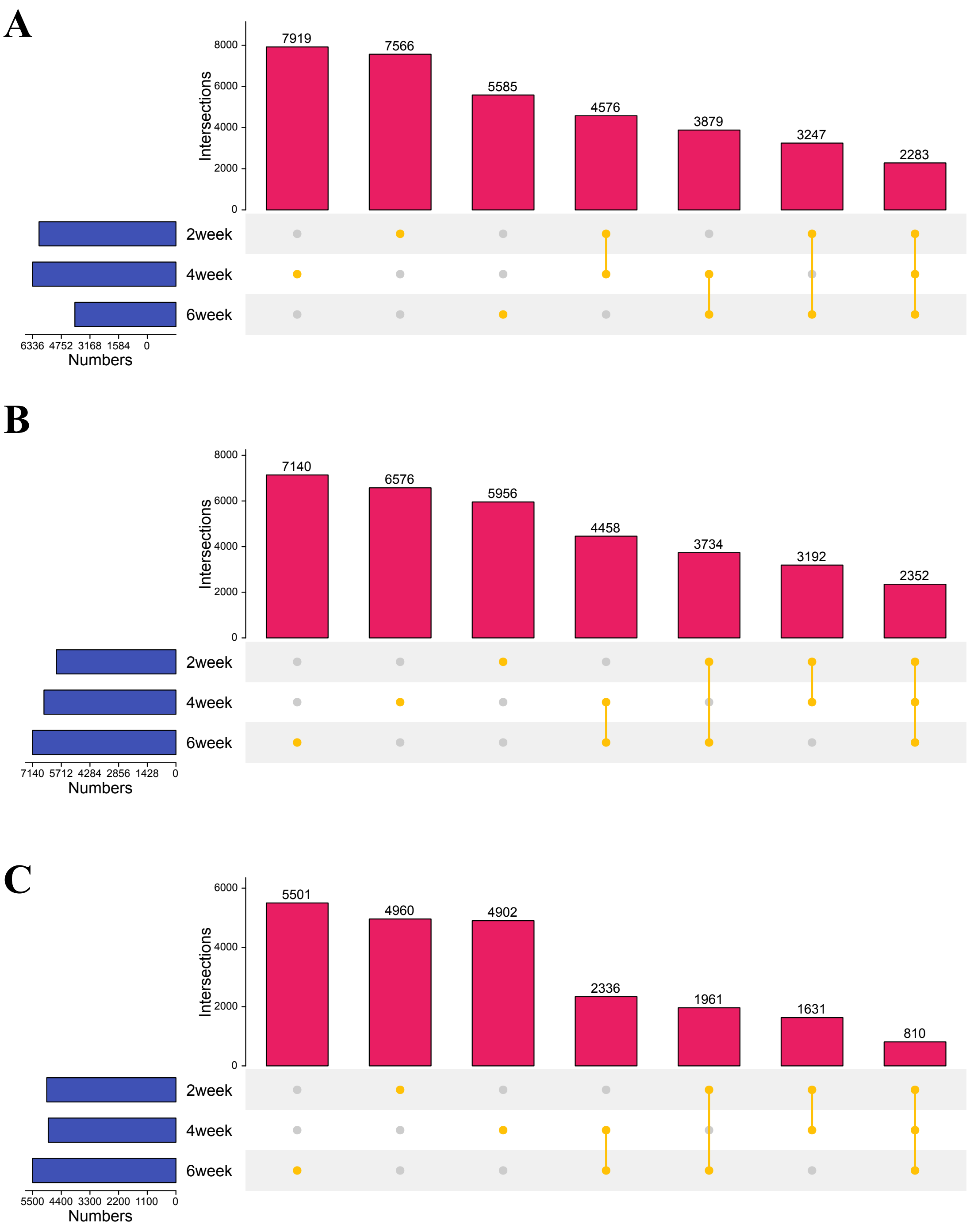

Fig. S6. The number of differentially expressed genes (padj<0.05) at dynamic development of breast muscle (A), liver (B) and fat (C) tissue in mallard and Pekin duck.

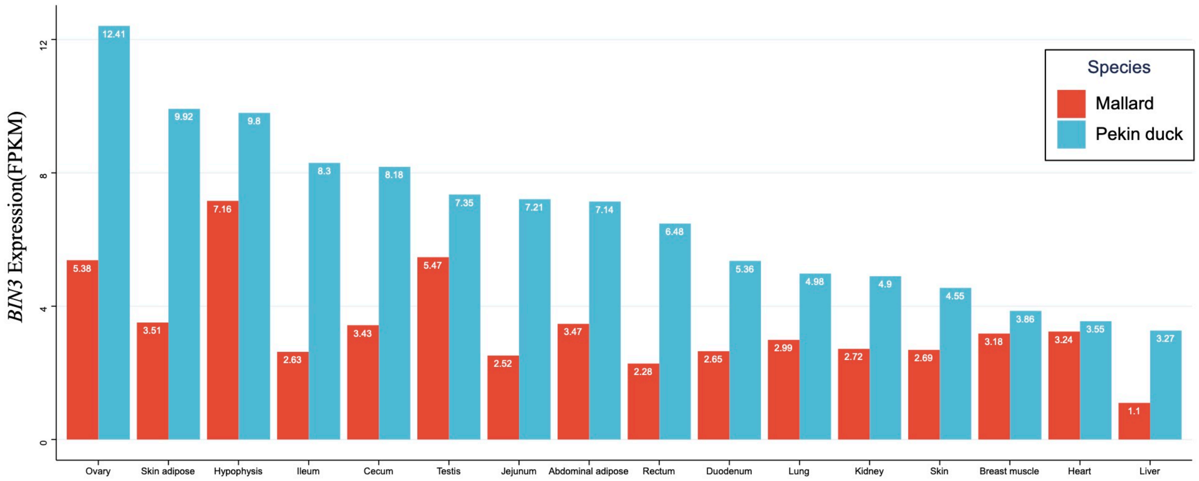

Fig. S7. The Expression profile of *BIN3* in 16 tissues of mallard and Pekin duck. *BIN3* was highly expressed in all 16 observed tissues in Pekin duck.

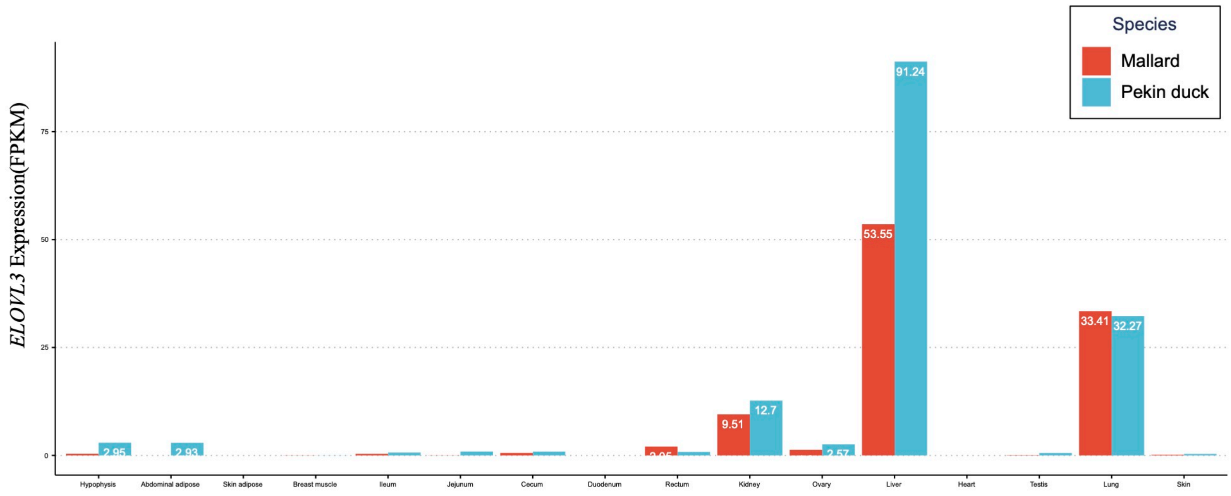

Fig. S8. The Expression profile of *ELOVL3* in 16 tissues of mallard and Pekin duck. *ELOVL3* was only highly expressed in liver and lung in Pekin duck.

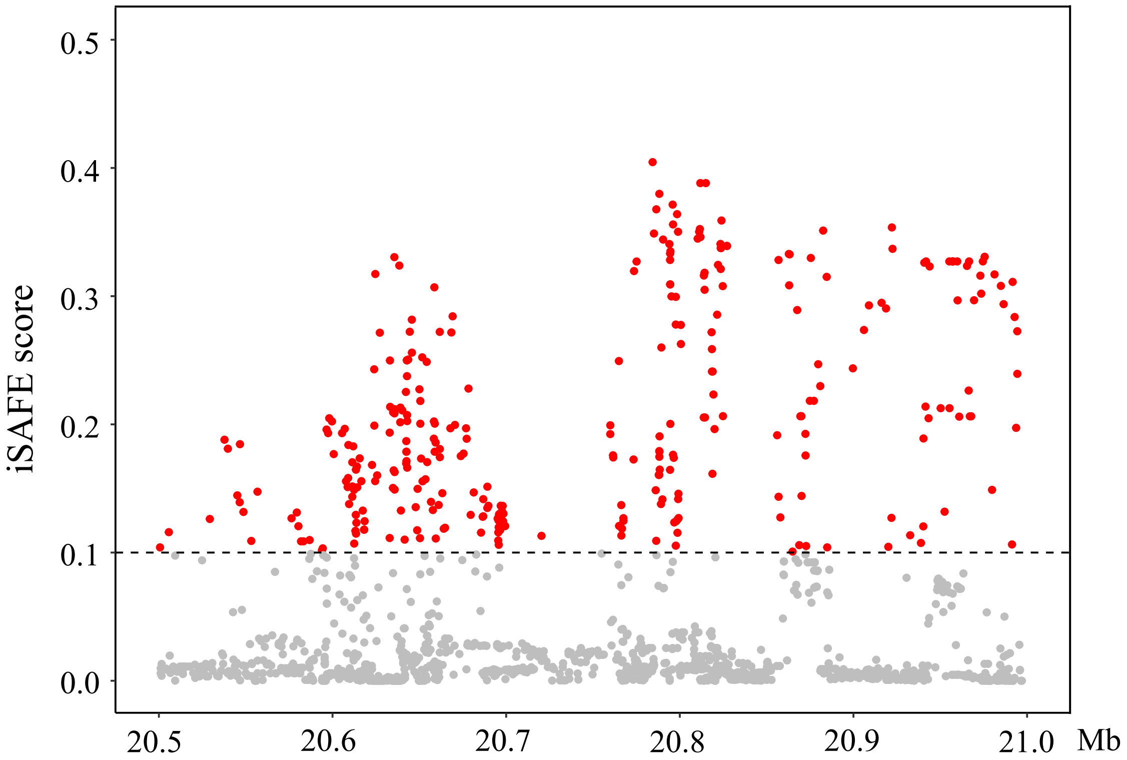

Fig. S9. Selective sweep regions arising from domestication found around the *ELOVL3* region on chromosome 7. Each dot in the iSAFE plots represents the average iSAFE score for 36 SNPs (step size 18 SNPs) in the region. The dashed line shows the empirical significance cut-off.

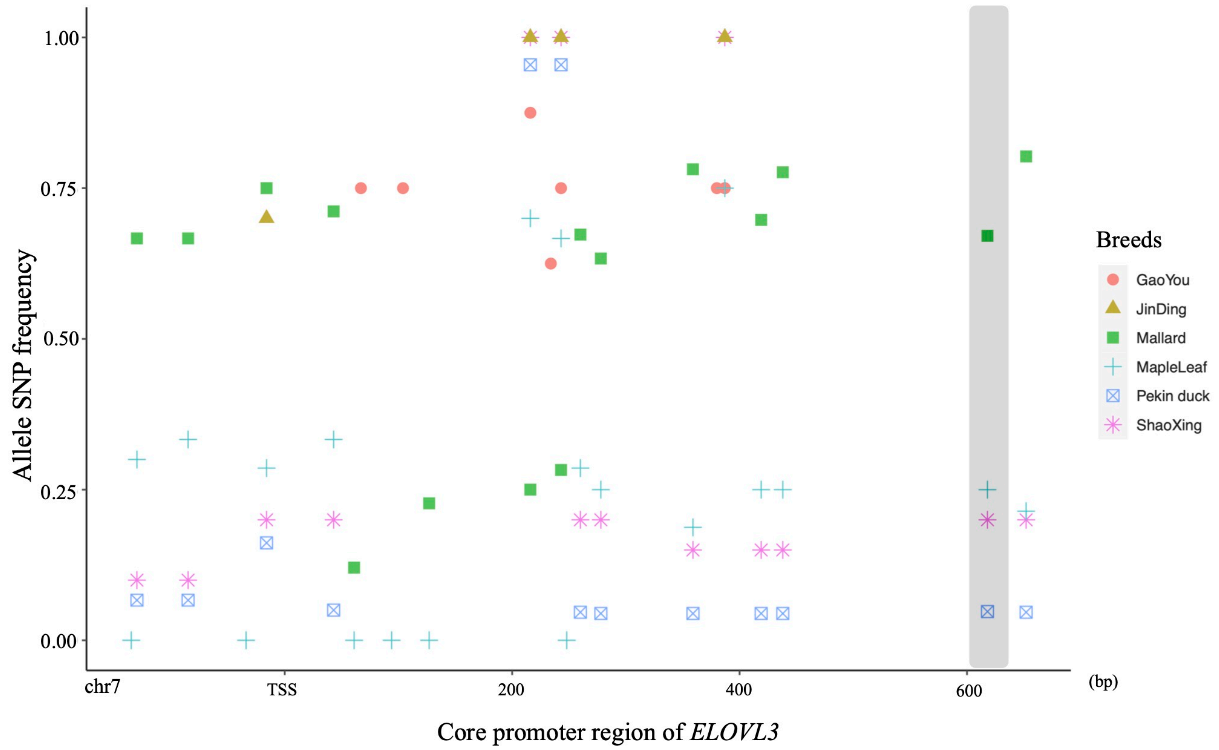

Fig. S10. The SNP sites and allelic frequencies of *ELOVL3* core promoter region in local duck breeds. The local duck breeds contained meat-type Pekin duck (MapleLeaf duck) and three Egg and dual-purpose type duck (ShaoXing duck, GaoYou duck and JinDing duck). Among the 4 local breeds, the key SNP (the upstream 619 site which is relative to the translation start site ATG) was detected in MapleLeaf duck and ShaoXing duck populations.

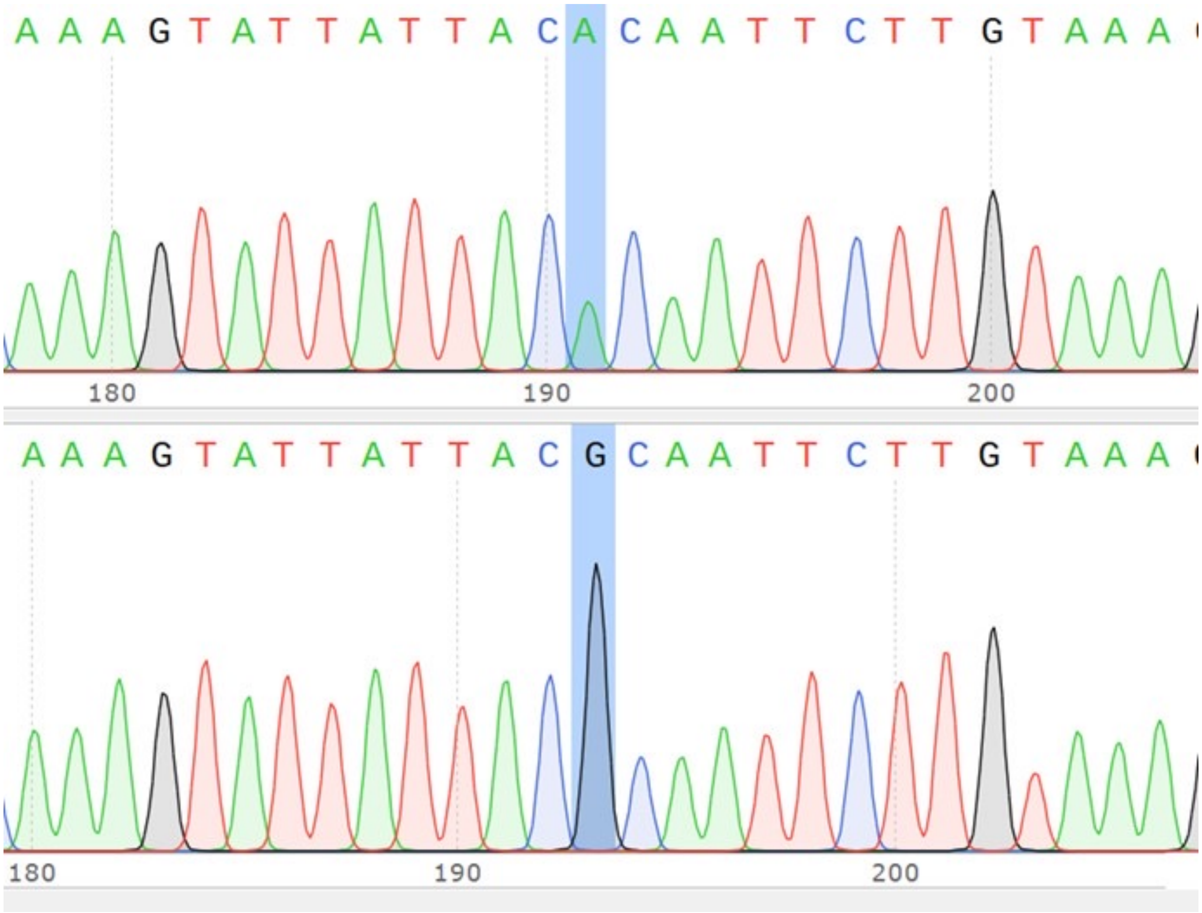

Fig. S11. Construction of mutation at site -619 (A<G) in the upstream regulatory region of the *ELOVL3* gene.

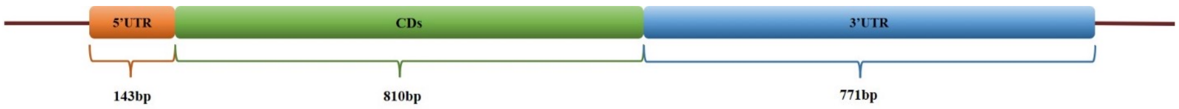

Fig. S12. Schematic diagram of duck *ELOVL3* cDNA structure. In this study, the complete cDNA sequence of duck *ELOVL3* gene was obtained by RACE technology, which contained a total of 1724 base pairs, of which the 5'UTR region contained 143bp (Orange); the CDs region was 810bp (Green); the 3'UTR region was 771bp (Blue).

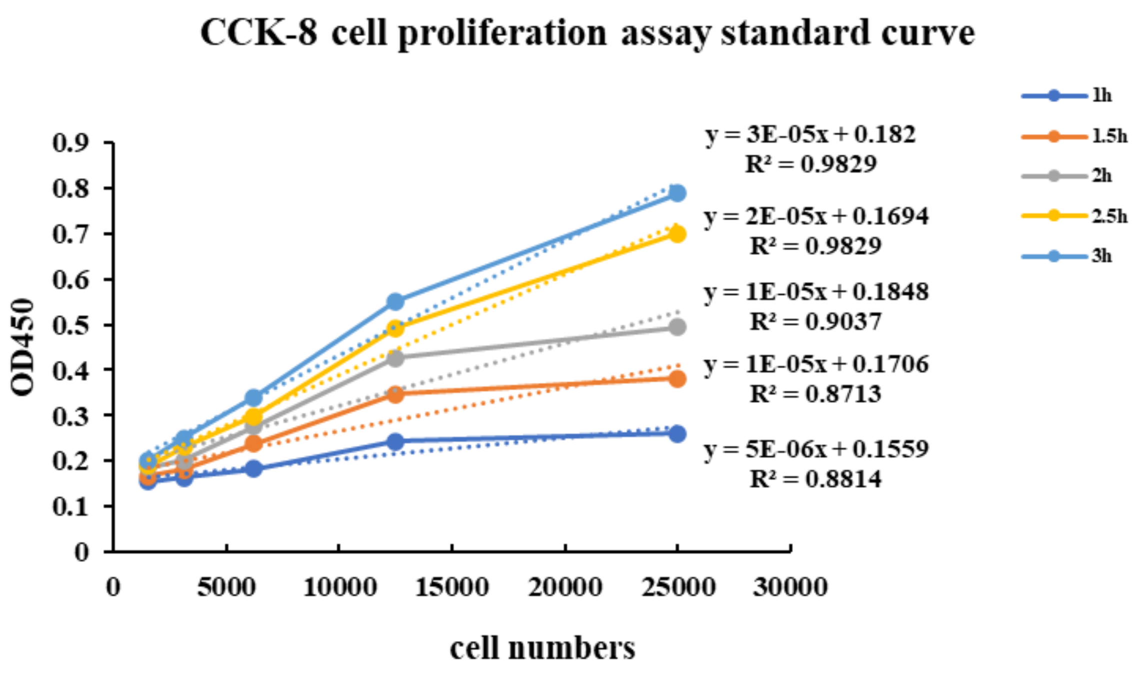

Fig. S13. CCK-8 Proliferation Assay Standard Curve for ICP1 Cells. The number of cells in the prepared ICP1 cell suspension was counted by a cytometer, and then diluted with culture medium in a gradient to form a cell concentration gradient, with six replicate wells in each group. After inoculation, the cells were cultured to make the cells adhere to the wall, and then CCK-8 reagent was added for a certain period of time to measure the OD value, and a standard curve with the number of cells as the abscissa (X axis) and the OD value as the ordinate (Y axis) was prepared. In this experiment, according to the standard curve, it was found that the seeding density of ICP1 cells should be between 1250-12500 cells per well, and CCK-8 was added and cultured for 2.5-3 hours. In this study, the cell seeding density was 4000 cells per well. After adding CCK-8 and culturing for 2.5 hours, the OD value at 450 nm was measured.

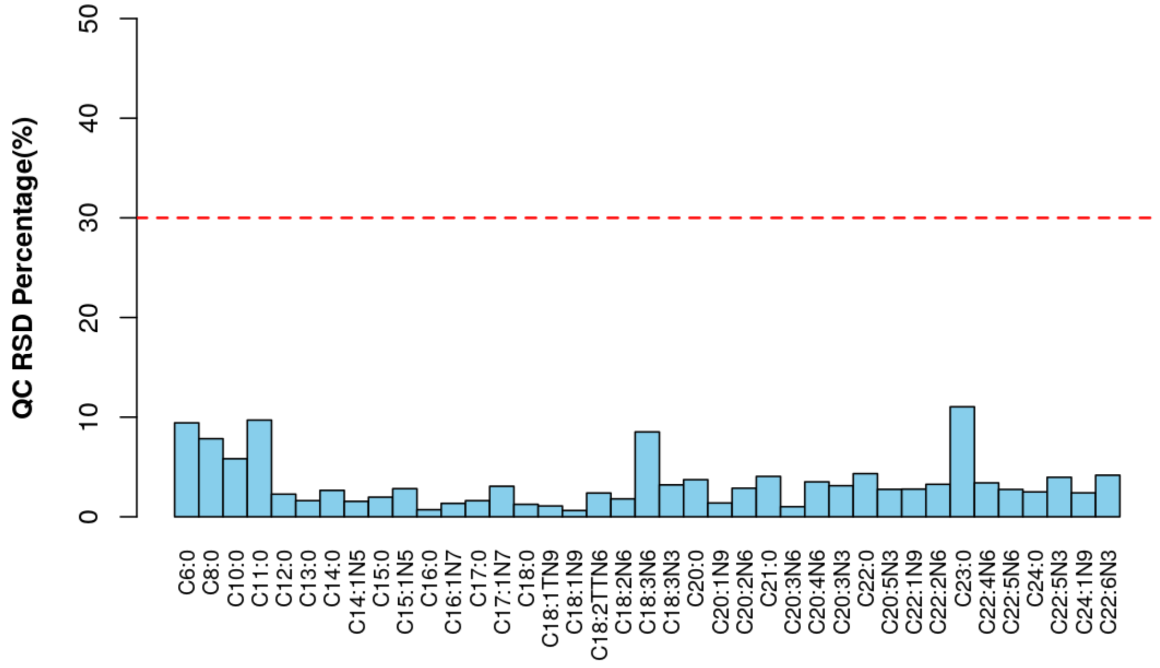

Fig. S14. Relative Standard Deviation (RSD) of within-batch QC samples. The relative standard deviation (RSD) of ion peak abundance in QC samples is an important indicator to reflect the quality of data. In this study, the RSDs were all less than 30%, indicating that the stability of the instrument was good.

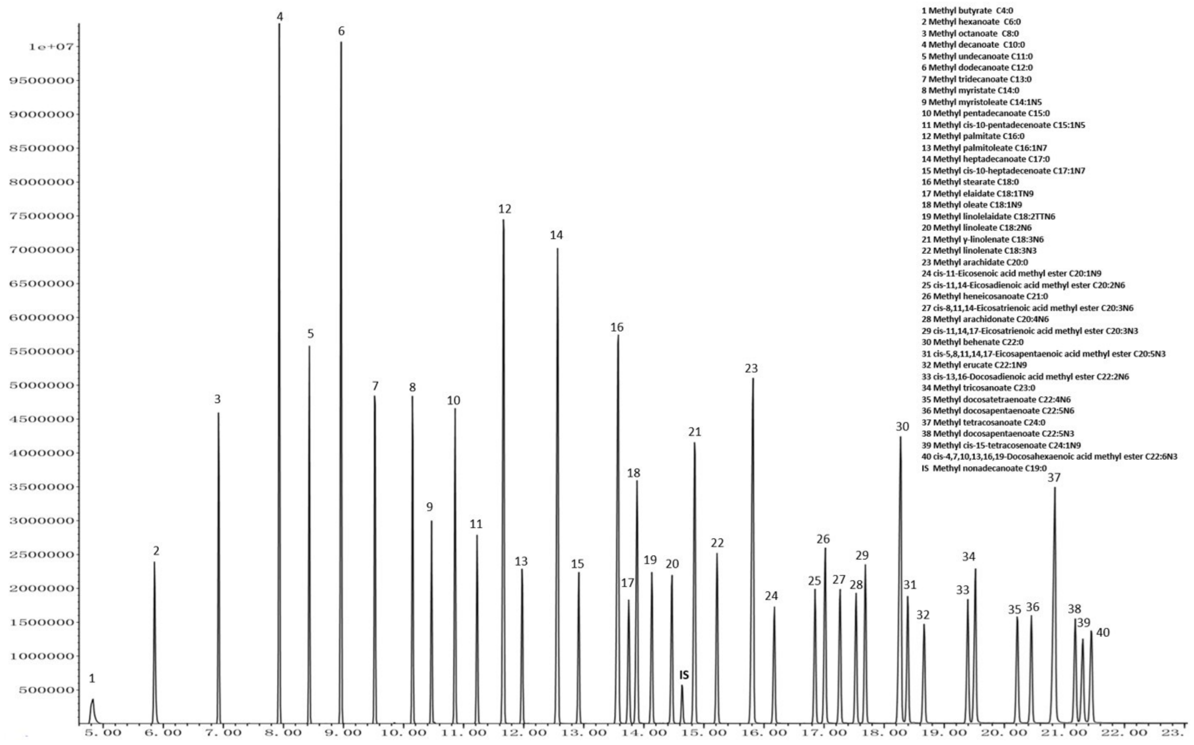

Fig. S15. Metabolite Standard Mixture TIC Profile. The TIC chart of the medium and long chain fatty acid standard is shown in the figure below. 40 kinds of fatty acid methyl ester standard products were analyzed by the established fatty acid analysis method, and a total of 40 kinds of fatty acids were obtained. It can be seen from the figure that the internal standard IS was separated from each standard product, and the chromatographic separation of each metabolite was good, and the peak shape was sharp and symmetrical, which could Mass Spectrometry quantification was performed for each metabolite.

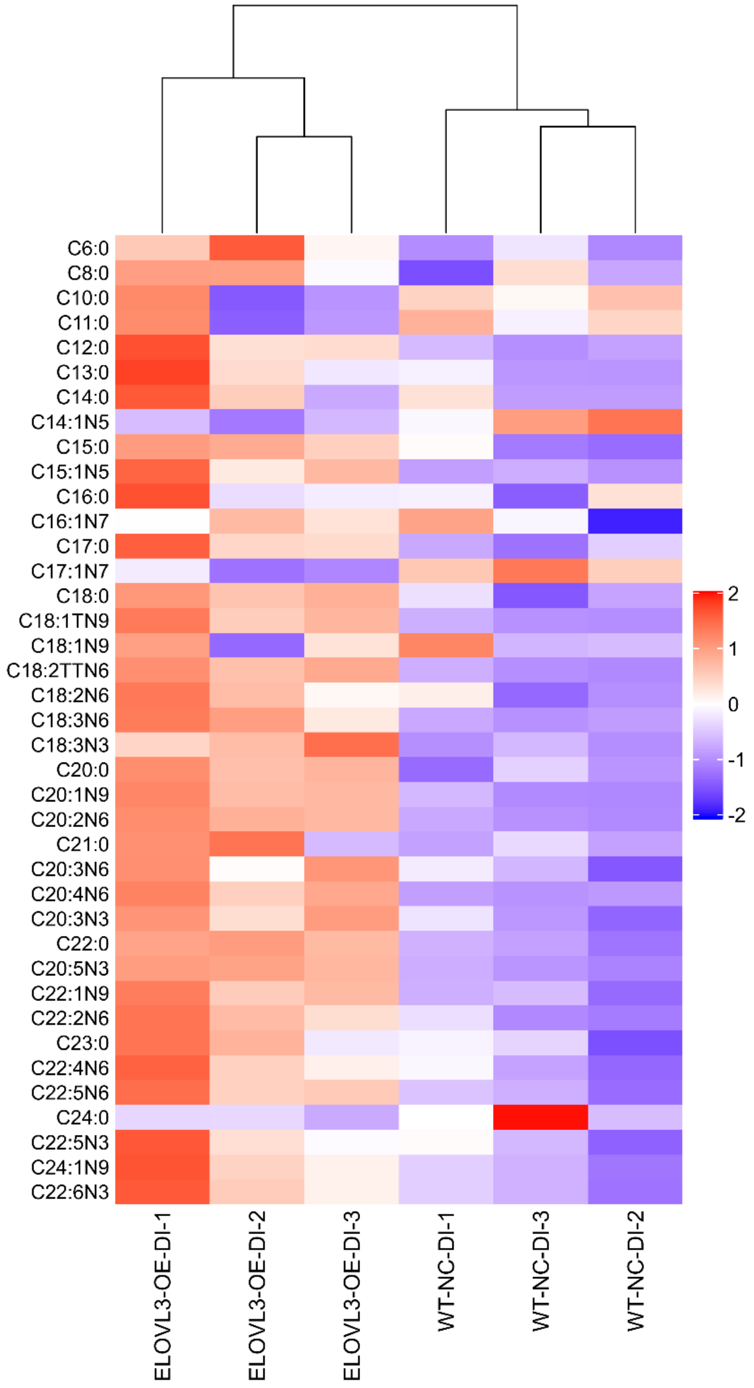

Fig. S16. Heatmap of biological replicates for fatty acid content determination by Gas Mass Spectrometry. The colors ranging from blue to red represent fatty acid content normalized with a z-score of -2 to 2, indicating low to high content. The sample clustering results are good, indicating good biological repeatability between samples.

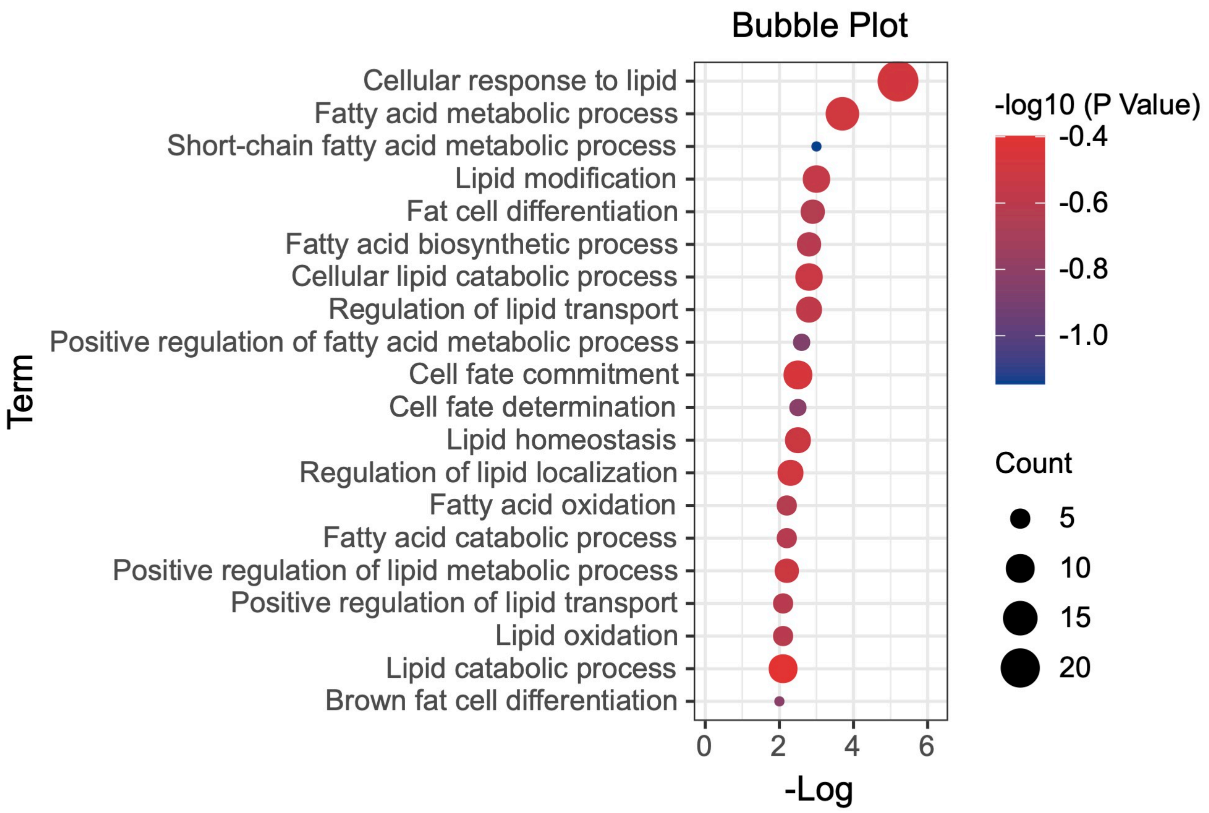

Fig. S17. The functional enrichment analysis to GO (Biology Process) of differentially expressed genes of ICP1 cells differentiation induced by overexpression of *ELOVL3*. Compared with wild type, these differentially expressed genes during cell differentiation were unique.
